# [2.2]Paracyclophane-Based Polyimides of Intrinsic Microporosity for Gas Separation

**DOI:** 10.1021/acsapm.6c00411

**Published:** 2026-03-31

**Authors:** Yuting Li, C. Grazia Bezzu, Anže Zupanc, Luis Simbari, Shrestha Banerjee, Dominik J. Kubicki, Tomislav Friščić, Marjan Jereb, Ross D. Jansen-van Vuuren, Mariolino Carta, Stefan Bräse

**Affiliations:** † Institute of Organic Chemistry (IOC), Karlsruhe Institute of Technology (KIT), Kaiserstraße 12, 76131 Karlsruhe, Germany; ‡ Department of Chemistry, Faculty of Science and Engineering, Swansea University, Swansea SA2 8PP, United Kingdom; § School of Chemistry, University of Birmingham, Edgbaston, Birmingham B15 2TT, United Kingdom; ∥ Faculty of Chemistry and Chemical Technology, University of Ljubljana, Večna pot 113, 1000 Ljubljana, Slovenia; ⊥ Instituto de Síntesis Química y Catálisis Homogénea, CSIC-Universidad de Zaragoza, C/Pedro Cerbuna 12, Facultad de Ciencias, Zaragoza 50009, Spain

**Keywords:** polyimide, [2.2]paracyclophane, gas separation, polymers of intrinsic microporosity, microporous, polymers

## Abstract

Polyimides (PIs) are a significant class of high-performance polymers due to their exceptional thermal, mechanical, and chemical stability. Their combination with polymers of intrinsic microporosity (PIMs) provides access to materials with structural robustness coupled with permanent microporosity. Progress in this area is frequently constrained by the limited availability of difficult-to-prepare and structurally complex dianhydrides. Building on our recent work on [2.2]­paracyclophane (PCP)-based PIMs for gas sorption, we report a series of PCP-polyimide PIMs synthesized from PCP-derived dianhydrides and amino-PCP monomers. The preparation of these dianhydrides enabled a systematic investigation of the monomer structure and connectivity. Two polymer families were obtained: PCP-PIs incorporating pseudo-*para* and pseudo-*meta*-PCP units and an ethanoanthracene analogue and a corresponding series of 4,4′-(hexafluoroisopropylidene)­diphthalic anhydride (**6FDA**)-based PCP polymers. Gas sorption measurements show very good CO_2_/N_2_ separation performance with the highest selectivity observed for the *meta*-PCP–ethanoanthracene system (**PCP-PI4**, ∼40). Polymers derived entirely from PCP-based dianhydride and bisaniline units in a pseudo-*meta* configuration also display a capability for strong separation of gases (CO_2_/N_2_ = 32.5), underscoring the role of monomer design in tuning the separation properties. Extensive structural and morphological characterizations were performed using multinuclear solid-state NMR, Fourier-transform infrared spectroscopy (FT-IR), wide-angle X-ray diffraction (WAXD), scanning electron microscopy (SEM), and energy-dispersive X-ray spectroscopy (EDX), while the thermal stability was determined by thermogravimetric analysis (TGA).

## Graphical abstract

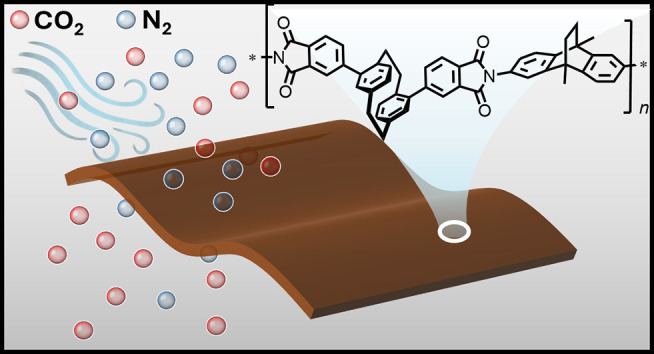

## 1 Introduction

Polyimides (PIs) are technologically important high-performance polymers due to their unique balance of thermal stability, mechanical strength, chemical resistance, and electrical insulation capabilities.^1^ Their exceptional stability in extreme environments including high temperatures, aggressive chemicals, and intense radiation, has made them indispensable in demanding sectors such as aerospace, electronics, and the automotive industry.^2^ Unlike most polymeric materials, polyimides remain structurally stable at temperatures exceeding 400 °C, exhibit excellent dielectric behavior, and display slow outgassing, releasing only minimal volatile molecules when exposed to high vacuum.^3^ These attributes make them especially well-suited for space technologies, microelectronics, and high-vacuum instrumentation.^4^ Consequently, polyimides are found in a wide range of applications including flexible printed circuits, wire insulation, structural adhesives, high-performance composites, and protective coatings,^5^ as well as tunable energy devices.^6^

Polymers of intrinsic microporosity (PIMs) constitute another notable class of materials defined by their rigid, contorted macromolecular backbones that hinder close chain packing, leading to permanent microporosity and significant free volume. This structural feature imparts exceptionally high surface areas and molecular permeability, making PIMs attractive for gas separation, adsorption, storage applications, and catalysis.^7^ However, apart from a few selected examples,^8^ they often suffer from limited mechanical strength and thermal stability that, after long exposure to gases, restrict their performance, especially under industrially demanding conditions.^9^

To overcome such limitations, a promising research direction involves the development of porous PIs that combine the intrinsic robustness of their backbone with tailored porosity. The introduction of pores increases surface area, lowers density, and enables selective molecular transport, opening opportunities in gas separation membranes, filtration, and energy storage devices.^10^ The rigid aromatic backbones of PIs provide excellent selectivity and thermal stability for separating gases such as CO_2_, O_2_, and H_2_,^11^ while high internal surface areas enhance performance in battery and supercapacitor electrodes.^12^ Additionally, their reduced thermal conductivity makes porous polyimides useful as lightweight insulators in aerospace or cryogenic systems. These design strategies expand polyimides beyond their traditional applications as passive coatings into roles as functional and active materials in energy and environmental technologies.^13^

Processing considerations also play a central role in PI applications. Soluble PIs offer key fabrication advantages, as they can be dissolved in common solvents and directly processed into films, fibers, or coatings, streamlining the manufacture of devices such as flexible displays, microelectronics, and protective encapsulation layers.^14^ Solubility further facilitates composite and blend formation with nanomaterials, enabling enhancements in properties such as toughness, conductivity, and barrier performance.^15^ Insoluble PIs, though more difficult to process, remain vital thanks to their superior durability, solvent resistance, and mechanical stability.^16^ Typically produced by curing soluble polyamic acid precursors, insoluble PIs exhibit outstanding resistance to thermal degradation and chemical stress, enabling unparalleled reliability in aerospace insulation, nuclear shielding, and high-voltage electrical systems.^4^

In this context, polyimides incorporated into polymers of intrinsic microporosity (PIM–PIs), either as homopolymers or via copolymerization, represent a particularly promising strategy for combining the chemical and thermal stability of PIs with the high free volume and intrinsic microporosity of PIMs.^17,18^ This unique combination allows one to overcome the robustness limitation by integrating microporosity into the polyimide framework, achieving a balance of high thermal resistance, chemical durability, and porosity-driven selectivity.^19,20^ These materials combine high gas transport efficiency with structural reliability, making them highly suitable for advanced separation membranes, catalyst supports, and electrochemical energy storage technologies, even when they are not soluble.^21,22^

In our recent work,^23^ we pioneered PIMs containing [2.2]­paracyclophane (PCP) units to understand how the rigidity and stability of these well-studied moieties would influence gas sorption. The work consisted of the synthesis of Tröger’s base (TB) PIMs containing PCPs, which were found to possess selectivity for CO_2_/N_2_ ranging from 46 to 70. The logical next step was to compare TB PCP PIMs with PCP-PI PIMs, since PI PIMs are well established in the literature.^24^ The first demonstration of PIMs constructed on a PCP framework has already highlighted the remarkable potential of this structural motif for gas separation applications. Building on this foundation, in this work, we report a series of PCP-based polyimide PIMs. In contrast to previously published systems that predominantly employ commercially available linear dianhydrides,^25^ our strategy utilizes PCP-derived dianhydrides **PCP-BA** in which an additional PCP scaffold was introduced to provide more rigidity and overall stability (Figure 1). In combination with the previously reported amino-PCP building blocks **PCP-** *p* **-DA** and **PCP-** *m* **-DA**, the reaction with **PCP-BA** afforded two polyimides, **PCP-PI1** and **PCP-PI2**, respectively. In addition, **PCP-BA** was copolymerized with dimethyl-ethanoanthracene-diamine (**EA**(**Me**)) to afford **PCP-PI4**.^18^ This comparative system was used to evaluate the influence of the PCP-based diamine units on the resulting polymer properties. For lateral structural extension, a commonly used linear 4,4′-(hexafluoroisopropylidene)­diphthalic anhydride (**6FDA**)^26–28^ was further employed and polymerized with **PCP-** *p* **-DA** and **PCP-** *m* **-DA**, yielding **PCP-PI3** and **PCP-PI5**, respectively, thereby enabling a direct comparison between PCP-based and conventional linear dianhydride systems.

**Figure 1:**
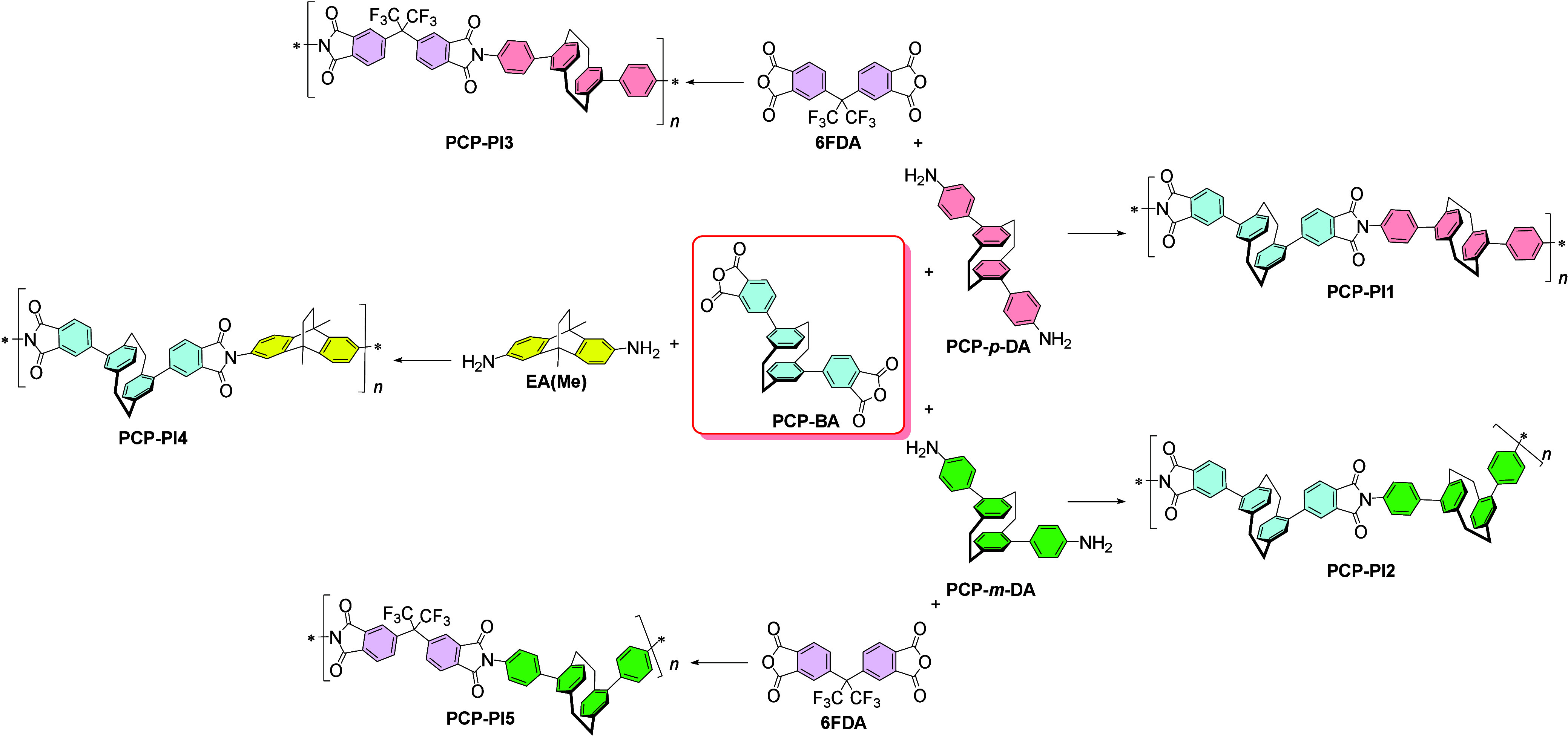
Chemical structures of the copolymeric PIMs studied in this work.

By systematically combining these monomers, we investigated the impact of incorporating PCP units into both the bisanhydride and the diamine components on the structure and properties of the resulting polyimides. This approach provides insight into the structural role of the PCP moiety within the polymer backbone and the influence of its orientation on polymer formation and material properties.

## 2 Experimental Section

Detailed information on the materials and methods is provided in the Supporting Information, where the full experimental procedures and compound characterization data are also described.

### 2.1 Synthesis Procedure of the PCP-BA

In a sealable vial, 4,15-dibromo[2.2]­paracyclophane (500 mg, 1.37 mmol, 1.00 equiv), dimethyl 4-(4,4,5,5-tetramethyl-1,3,2-dioxaborolan-2-yl)­benzene-1,2-dicarboxylate **2** (1.09 g, 3.41 mmol, 2.50 equiv), Pd­(PPh_3_)_4_ (78.9 mg, 68.3 μmol, 0.05 equiv), and potassium phosphate (562 mg, 4.10 mmol, 3.00 equiv) were dissolved under argon atmosphere in 20 mL of dioxane and 5 mL of H_2_O. The mixture was heated to 90 °C for 16 h and then cooled to 22 °C. The mixture was extracted with dichloromethane (DCM), and the solvent was removed under reduced pressure. The crude solid was purified by flash column chromatography (silica, hexane/ethyl acetate 10:1) to obtain the title product tetramethyl 4,4′-(1,4­(1,4)-dibenzenacyclohexaphane-12,42-diyl)­diphthalate **4** (610 mg, 91% purity, 937 μmol, 69% yield) as an off-white solid. Then, to a 100 mL round-bottom flask equipped with a magnetic bar and a refluxing condenser, **4** (500 mg, 844 μmol, 1.00 equiv) and KOH (1.89 g, 33.7 mmol, 40.0 equiv) were added to tetrahydrofuran (THF) (16.0 mL) and water (24.0 mL). The mixture was stirred at 80 °C in an oil bath for 22 h. After cooling to 25 °C, the organic layer was evaporated, and the remaining aqueous phase was acidified by hydrochloric acid (2 M) to pH = 5. The precipitate was filtered off and dried under high vacuum to afford the title product 4,4′-(1,4­(1,4)-dibenzenacyclohexaphane-12,42-diyl)­diphthalic acid **5** (423 mg, 788 μmol, 93% yield) as a colorless solid. Then **5** was dehydrated in excess of acetic anhydride for 3 h. The excess acetic anhydride was evaporated under vacuum, and then, the crude product was washed with DCM and acetone. The off-white solid was dried under vacuum to yield the product 4,15-bis­(5,5′(isobenzofuran-1,3-dione))[2.2]­paracyclophane **PCP-BA** (750 mg, 1.50 mmol, 67% yield) as a pale green solid.

### 2.2 General Procedure of Polymer Synthesis

The bis-anhydride was dissolved in ethanol in a two-necked flask equipped with a Dean–Stark apparatus and reflux condenser under a nitrogen atmosphere. Triethylamine was added, and the mixture was refluxed for 1 h. The side arm was opened to remove the solvent under a stream of nitrogen to give a highly viscous solution.

The Dean–Stark trap was filled with toluene before half the amount of solvent, N-Methyl-2-pyrrolidone (NMP):toluene (4:1 mixture), and the diamine were added, followed by another aliquot of solvent. The reaction mixture was heated at 80 °C for 1 h, and then, the temperature gradually increased to 200 °C. The reaction was maintained at this temperature until the desired viscosity was achieved. The mixture was cooled to room temperature and diluted with chloroform. The mixture was poured into ethanol to precipitate a solid. The solid was collected by filtration and washed with ethanol until the washings were clear. Chloroform was added to the resulting solid followed by methanol, and the resulting gel/solid was filtered off. The latter treatment was repeated, and then, the powder was refluxed in methanol for 24 h, filtered, and then dried in a vacuum oven at 100 °C for 8 h to afford the desired polymer. Detailed synthetic procedures for each individual polymer are given in the Supporting Information.

## 3 Results and Discussion

### 3.1 Synthesis of the Monomer and Polymer

To obtain the corresponding polyimide, the PCP-bis-aniline precursors **PCP-** *p* **-DA** and **PCP-** *m* **-DA**, prepared according to our previous work,^29^ were copolymerized with the innovative bis-anhydride monomer **PCP-BA** (Figure 1). This bis-anhydride monomer required a dedicated synthetic route, commencing with the preparation of an *ortho*-carboxylated intermediate **5** via a two-step sequence involving the preparation of boronic ester **2**, which was cross coupled with dibromoparacyclophane **3** (Scheme 1). Subsequent dehydrative cyclization of this key intermediate furnished the targeted bis-anhydride **7**. To mitigate the inherently low solubility of bis-anhydride structures,^30^ a pseudo-*meta*-substituted PCP scaffold was deliberately selected (**7**); this substitution pattern provides markedly improved solubility compared to the pseudo-*para* analogue.

**Scheme 1:**
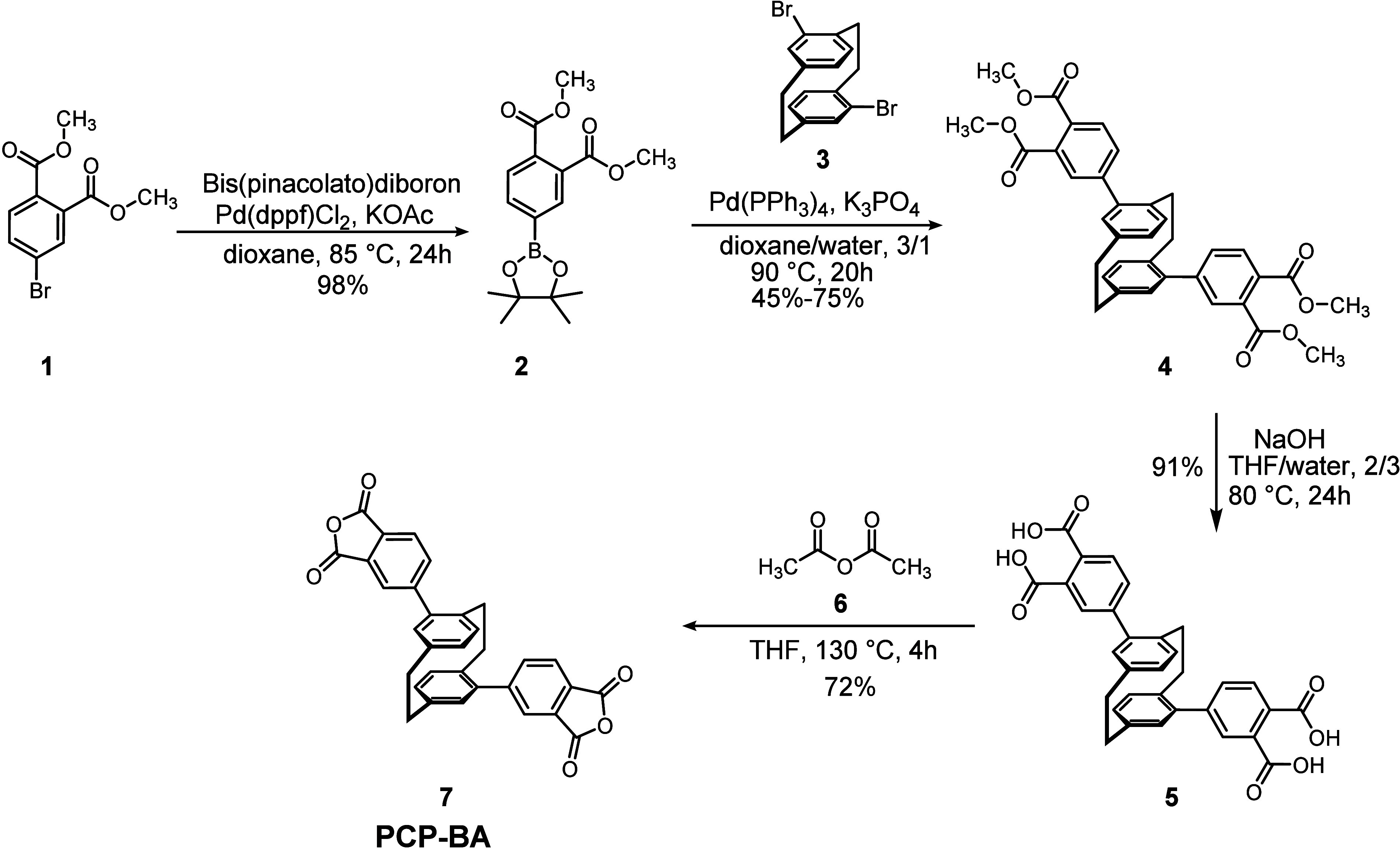
Synthesis of the PCP-Based Dianhydride **7**

Using the conditions shown in Scheme 2, we synthesized a series of polyimides, all incorporating the PCP unit as the central structural motif. Our approach began with the combination of the two main PCP-based building blocks: **PCP-BA** and **PCP-DA**.

**Scheme 2:**
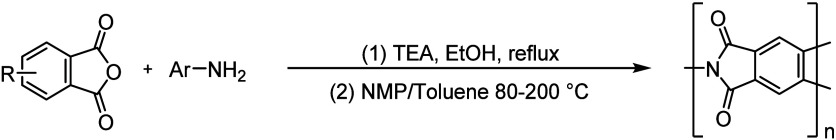
Synthesis of PCP-PIs via a One-Pot Ester–Acid Route^17^

As a representative example, **PCP-PI3** was synthesized using a well-established “*one-pot*” method based on the ester–acid route for polyimide preparation.^17^ In this approach, the synthesis starts with the conversion of the dianhydride monomer into a diester-diacid intermediate via solvolysis with an aliphatic alcohol, typically ethanol, in the presence of a tertiary amine catalyst such as triethylamine (TEA). After the removal of excess ethanol and TEA by distillation, a viscous diester-diacid intermediate was obtained. Next, corresponding diamine monomer is added to a high-boiling solvent, *N*-methyl-2-pyrrolidone (NMP), and toluene as an azeotropic agent. The reaction mixture was heated to 160–200 °C during which rapid poly­(amic acid) formation occurred, followed by cyclodehydration to afford the final polyimide. The combination of an elevated temperature and efficient removal of water through azeotropic distillation promotes effective chain growth and a high degree of imidization in the homogeneous solution. All other PCP-based polyimides were synthesized following this general procedure with only minor adjustments to reaction times depending on the specific monomer combinations. Detailed synthetic procedures and characterization data are provided in the Supporting Information.

### 3.2 Characterization of the Polymers

First, the resulting polyimides were characterized by Fourier-transform infrared spectroscopy (FT-IR) to confirm the successful polymerization and formation of the imide structure. This technique allows for monitoring of the disappearance of characteristic absorption bands belonging to the monomeric precursors and the emergence of signals associated with the polymer backbone.

A representative example is shown in Figure 2, where the spectra clearly illustrate the chemical transformation from the monomers to the final polyimide. The amine stretching vibrations observed in the spectrum of the **PCP-** *m* **-DA** monomer (Figure 2b) completely disappear in the corresponding polyimide spectrum (Figure 2c), indicating that the amine groups reacted fully with the anhydride moieties. At the same time, the carbonyl stretching bands of the bis-anhydride precursor (Figure 2a), which typically appear at higher wavenumbers (around 1850 and 1780 cm^–1^), shift to lower values (approximately 1775 and 1720 cm^–1^) in the polyimide. This shift is characteristic of the conversion of anhydride groups into imide carbonyls, confirming the successful formation of the imide rings. Additional characteristic absorptions for the imide structure were also observed, such as the C–N stretching band around 1360 cm^–1^ and the imide ring deformation band near 720 cm^–1^. Together, these features provide clear spectroscopic evidence of the completion of the imidization reaction and the formation of the desired polyimide network.

**Figure 2:**
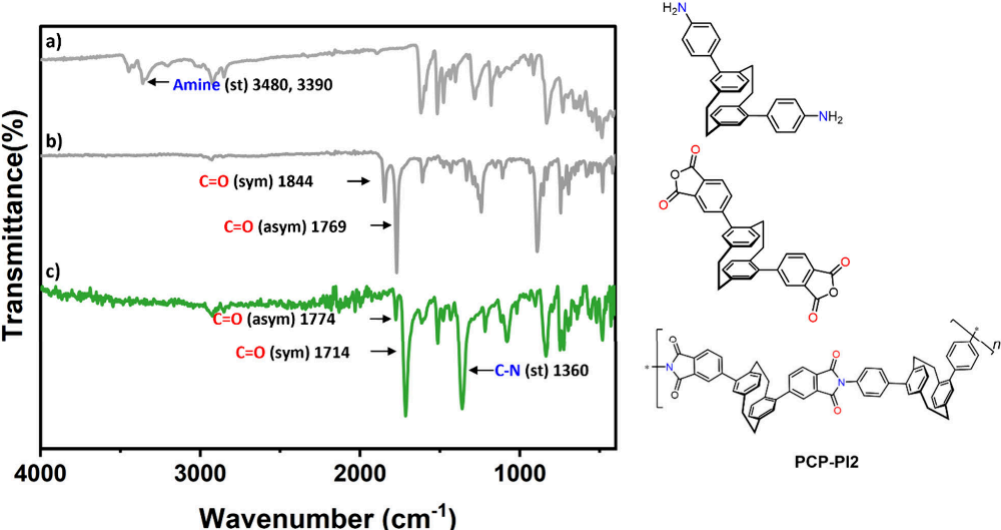
FT-IR of the overlay of (a) **PCP-** *m* **-DA** (dianiline), (b) **PCP-BA** (bisanhydride), and (c) **PCP-PI2** (polyimide), showing the most significant spectral bands.

All other PCP-based polyimides exhibited similar FT-IR spectra, showing the same set of characteristic imide bands and the disappearance of the monomeric signals along with the typical signals of the −CF_3_ moieties in the **6FDA-PIs** and the extra aliphatic moieties that belong to the EA­(Me). These consistent results confirm the successful synthesis and structural integrity of the entire series of PCP-containing polyimides.

To further verify the chemical structure and successful synthesis of the polyimides, ^1^H NMR spectroscopy was performed on **PCP-PI2**, **PCP-PI3**, and **PCP-PI4**. The spectra exhibited the expected broad signals characteristic of polymeric materials, confirming the presence of multiple proton environments along the polymer backbone. Integration of the signals corresponded well with the proposed chemical structures. In all cases, characteristic resonances of the PCP scaffold were observed with methylene protons appearing in the range of 3.27–2.71 ppm, accompanied by aromatic proton signals associated with the various aromatic moieties, typically distributed between 8.13 and 6.77 ppm. These observations collectively support the successful synthesis and the expected structural features of the target polyimides.

With poor solubility of some of the polymers, we carried out solid-state Magic Angle Spinning (MAS) NMR experiments to characterize their atomic-level structure and determine their purity. Figure 3 shows the ^13^C, ^15^N, ^19^F, and ^1^H MAS NMR spectra of the materials. In all materials, the ^13^C spectra contain a set of resonances that can be attributed to the carbonyl (166–167 ppm), aromatic (110–150 ppm), and aliphatic (10–50 ppm) local environments. The signal at 132 ppm and the region between 34 and 35 ppm in all samples possibly correlate to carbon atoms of benzene rings and methylene carbons of the *para*-cyclophane moiety.^29^ The NMR spectra of **PCP-PI3** and **PCP-PI5** materials also exhibit distinct C­(CF_3_)_2_ environments around 65 ppm. The positioning of the signal corresponding to the phthalimide carbonyls is influenced by the vicinity of the −CF_3_ substituents and is found to be 167 ppm for **PCP-PI1**, **PCP-PI2**, and **PCP-PI4** and 166 ppm for **PCP-PI3** and **PCP-PI5**. For the **PCP-PI4** material, there are two additional signals, labeled as C_quat_ and CH_3_, which we attribute to the quaternary and methyl groups, respectively, of the norbornane moiety. The ^13^C-resonance of the CF_3_ groups is typically expected to appear in the range of 115–130 ppm, which means that the corresponding signals are masked by the signals of the aromatic species in the polymers.

**Figure 3:**
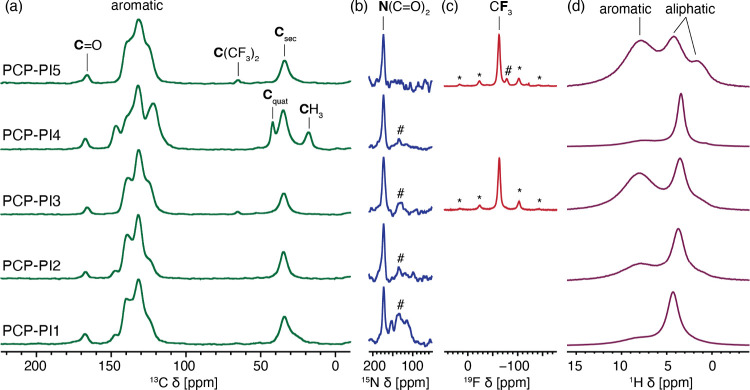
Solid-state MAS NMR characterization of the polymers **PCP-PI1**–**PI5** at 9.4 T and room temperature: (a) ^13^C CP at 15 kHz MAS, (b) ^15^N CP at 8 kHz MAS, (c) ^19^F echo at 15 kHz MAS, and (d) ^1^H echo at 8 kHz MAS. Asterisks (*) indicate spinning sidebands (these are asymmetrical due to Chemical Shift Anisotropy (CSA), which is related to the fact that in solids the chemical shift depends on the crystallite orientation relative to the external magnetic field. This distribution of chemical shifts is, in general, not symmetric around the central peak, and the spinning sideband manifold reflects this distribution). Hash symbols (#) indicate an unidentified impurity, likely NMP trapped into the pores. C_sec_ and C_quat_: secondary and quaternary carbons, respectively.^31^

The ^1^H NMR spectra are dominated by broad signals with maxima at around 3.5 and 7.5 ppm that correspond to protons connected to aliphatic and aromatic moieties, respectively. The ^15^N spectra all exhibit a sharp signal at about 173 ppm, corresponding to the amide nitrogen of the phthalimide moiety. Notably, ^15^N NMR also indicates the presence of small amounts of other nitrogen-containing species, which we attributed to NMP residues immobilized in the pores of the polymers.^32^ This interpretation is consistent with the presence of a broad shoulder on the carbonyl signal extending up to 180 ppm in the ^13^C spectrum. The ^19^F spectra of the **PCP-PI3** and **PCP-PI5** materials also exhibit a signal at ca. −63 ppm, corresponding to the CF_3_ groups on the polymer. An additional low-intensity signal (∼10% of the total integrated intensity) is present in the ^19^F spectrum of **PCP-PI5** at −78 ppm, which we attribute to −CF_3_ groups in an impurity phase.^33^ All individual solid-state spectra are provided in Figures S3 and S19 in the Supporting Information.

The MALDI-TOF spectrum of the most soluble polymer, **PCP-PI2**, after chloroform washing under ultrasound, showed a pattern of repeat units with a *M* ≈ 854 difference between consecutive maxima (Figure 4). The result is consistent with a polymeric structure composed of heterodimer units. The mass spectrometry data indicates an oligomer size of ca. 5 units, a footprint of polymerization, that could be a result of poor sensitivity of the measurement but also polymer cleavage during sonic treatment.

**Figure 4:**
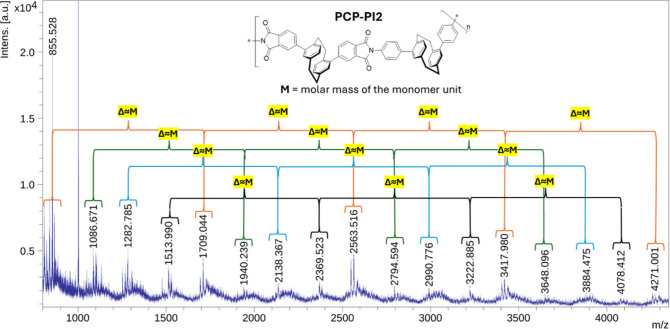
MALDI-TOF spectrum of repeating units of **PCP-PI2**.

### 3.3 Gas Adsorption Assessment and Textural Properties

All of the synthesized polyimides were evaluated for their gas adsorption behavior using both CO_2_ and N_2_ as probe gases, as is standard for microporous polymers. Nitrogen adsorption is generally used to measure surface area and pore volume, although CO_2_ adsorption isotherms at 273 K often gives a more accurate picture when pores are very small (ultramicropores), because its smaller kinetic diameter allows it to access narrower voids that N_2_ might not penetrate efficiently.^34^

The surface areas and total pore volumes were determined via Grand Canonical Monte Carlo (GCMC) analysis of the CO_2_ adsorption at 273 K but also via BET calculation from CO_2_ adsorption at 195 K. The latter could be considered more appropriate, as the saturation pressure of CO_2_ at 195 K is 1 bar, making the BET calculation more accurate within the relevant range. On the other hand, the calculation made from the adsorption at 273 K (despite being very reliable) may lead to underestimation of the surface area, as at 1 bar we are still far away from the saturation pressure, and the calculation is done while the polymer is still adsorbing CO_2_.^35^ Reporting assessments at both temperatures gives a broader picture of the overall porosity.

Table 1 summarizes the results obtained for the PCP-based polyimides (PCP-PIs) synthesized in this work. The data show that the two polymers derived from both monomers incorporating the PCP core exhibit comparable surface areas. Among them, **PCP-PI2** (335 m^2^g^–1^), in which both PCP units possess a pseudo-*meta* conformation, was found to be slightly more porous than **PCP-PI1** (302 m^2^g^–1^), where the bisanhydride moiety is pseudo-*meta* and the PCP-diamine (PCP-DA) pseudo-*para*. However, considering the inherent uncertainty associated with gas adsorption measurements, these differences fall within experimental error, and the two materials can therefore be regarded as having very similar porosities.

**Table 1:** Textural Properties of the PCP-PIs

Polymer	Surface Area m^2^g^–1^ ^a^ (m^2^g^–1^)^b^	Total Pore Volume cm^3^g^–1^ ^a^ (cm^3^g^–1^)^b^	Solubility	*Q*_st_ kJ mol^–1^ ^c^	IAST Selectivity^d^
**PCP-PI1**	302 (285)	0.2814 (0.0918)	Poor	40.7	27.5
**PCP-PI2**	335 (278)	0.2925 (0.0913)	DMAc (50 °C)	30.8	32.5
**PCP-PI3**	281 (240)	0.2932 (0.0794)	DMAc (50 °C)	38.9	25.5
**PCP-PI4**	400 (330)	0.3851 (0.1068)	CHCl_3_ (partly)	35.5	39.9
**PCP-PI5**	285 (250)	0.2783 (0.0908)	Poor	34.2	27.8

a From CO_2_ at 195 K.

b From CO_2_ at 273 K.

c Isosteric heat of adsorption from isotherms collected at 273 and 298 K, fitted with the Langmuir–Freundlich equation and calculated via the Clausius–Clapeyron equation.

d IAST selectivity, calculated for a hypothetical mixture CO_2_/N_2_ of 15/85 at 298 K.

The two 6FDA-containing polyimides, namely, **PCP-PI3** and **PCP-PI5**, also exhibited closely comparable surface areas (∼281–285 m^2^g^–1^), slightly lower than for the other PCP-PIs. Such behavior is not unexpected, as the 6FDA monomer, which, despite containing the bulky −CF_3_, generally used to increase free volume, introduces a higher degree of flexibility into the polymer backbone, potentially allowing for tighter chain packing in the solid state and consequently reducing accessible free volume. Among all samples, **PCP-PI4** displayed the highest surface area with 400 m^2^g^–1^. This result is consistent with the presence of the structural unit, as EA­(Me)-based moieties are well-known to promote enhanced microporosity and high surface areas in polymers of intrinsic microporosity (PIMs).^18,36^

It is also very important to evaluate the overall CO_2_ uptake of the materials at both 273 and 298 K, as these measurements provide valuable insight into their potential for carbon capture applications such as the removal of CO_2_ from flue gas streams (the typical flue gas compositions contain approximately 15% CO_2_ and 85% N_2_, along with trace amounts of other gases, water vapor, and acidic impurities such as SO_*x*_ and NO_*x*_). Assessing adsorption at these two temperatures not only helps to evaluate the intrinsic uptake of CO_2_ under conditions relevant to industrial processes but also enables the estimation of the isosteric heat of adsorption (*Q*_st_). This thermodynamic parameter provides an indication of the strength of interaction between the CO_2_ molecules and the polymer framework, which is crucial for balancing high adsorption capacity with efficient regeneration (i.e., desorption after capture). Our results show that *Q*_st_ for these polyimides varies in a range of 30–40 kJ mol^–1^, suggesting that the main adsorption mechanism is predominantly governed by physisorption, although a slight contribution from chemisorption cannot be entirely ruled out.

The overall CO_2_ adsorption, which is an important factor to assess carbon capture potential, was found to correlate well with the porosity characteristics. The EA­(Me)-based polyimide exhibited the highest CO_2_ uptake, consistent with its larger surface area and higher microporosity. In contrast, the PCP-PCP-based polyimides displayed moderate adsorption capacities but were still in a good range, and the 6FDA-containing polymers showed the lowest CO_2_ uptakes within the series. This trend is in line with expectations, as the greater chain flexibility imparted by the 6FDA units likely promotes denser molecular packing, thereby reducing the accessible free volume available for gas adsorption.

To further evaluate the gas separation potential of the synthesized polyimides, Ideal Adsorbed Solution Theory (IAST) calculations were employed.^37,38^ The IAST approach provides a valuable means of predicting the selectivity of a material toward different gases in binary mixtures, based solely on single-component adsorption isotherms,^39^ and is particularly useful for powder samples, where direct mixed-gas adsorption experiments are challenging due to experimental complexity, limited material availability, or scarce solubility that prevent assessment via other techniques. Our results indicate that the polymer with the highest surface area in the series also exhibits the highest predicted CO_2_/N_2_ selectivity with a value of ∼40 (Table 1). The other polyimides display relatively similar but somewhat lower selectivities with the pseudo-*meta*/pseudo-*meta* **PCP-PI2** showing a slightly higher value of 32.5 compared to the related pseudo-*para*/pseudo *meta* **PCP-PI1**, which confirms that the pseudo-*meta* position gives both higher porosity and higher selectivity and that the appropriate design of these monomers, indeed, helps tailor the performance.

The 6FDA-based PCP-PIs showed moderate selectivity values (25.5–27.8), aligned with those of the PCP-polyimides reported by Jiang and co-workers in their recent paper,^25^ confirming the validity of our methods. Although such selectivity values seem moderate, they demonstrate that the innovative polyimide design contributes to good CO_2_/N_2_ separation performance at 1 bar and room temperature.

To place our results in context, it is even more useful to compare them with those of related polyimide-based PIMs reported in the literature. In a recent study, the group led by Ma^28^ described a series of PCP-6FDA polyimides exhibiting BET surface areas of approximately 430 m^2^ g^–1^ and CO_2_/N_2_ selectivities around 23, values that are closely aligned with those obtained in the present work. Earlier pioneering contributions by Budd and McKeown^40^ on polyimide-based PIMs reported BET surface areas in the range of 480–700 m^2^ g^–1^ with CO_2_/N_2_ selectivities of approximately 23. In a subsequent study, the same group reported PIM–PIs displaying surface areas of 470–680 m^2^ g^–1^ and selectivities between 22 and 26, further establishing the characteristic balance between microporosity and gas separation performance in this materials class.^41^ More recently, Sivaniah and co-workers reported polyimides incorporating carboxylic acid and tertiary amine functionalities with BET surface areas spanning 55–339 m^2^ g^–1^ and CO_2_/N_2_ selectivities in the range of 19–30.^42^ Another highly active group in this field, led by Professor Pinnau, has reported several PIM-polyimides based on substituted spirobisindane moieties.^43^ These materials exhibited BET surface areas between 190 and 243 m^2^ g^–1^ and CO_2_/N_2_ selectivities of 23–30, with the highest selectivities typically observed after physical aging, where overall adsorption decreased but selectivity increased. In a related study, the same group described plasticization-resistant polyimides with surface areas of 260–450 m^2^ g^–1^ and CO_2_/N_2_ selectivities ranging from 12 to 20.^44^

Collectively, the performance metrics of our materials fall well within the range reported for state-of-the-art polyimide-based PIMs, confirming that the present systems achieve a comparable balance between the intrinsic microporosity and gas separation selectivity.

Overall, the outlined observations suggest that these materials hold promising potential for carbon capture applications, particularly considering that further structural optimization could further enhance their selectivity and that films potentially produced from **PCP-PI2** and **PCP-PI3** may show enhanced gas separation properties. Figure 5a presents the CO_2_ adsorptions at 273 K, and Figure 5b presents the corresponding IAST selectivity for all synthesized polyimides.

**Figure 5:**
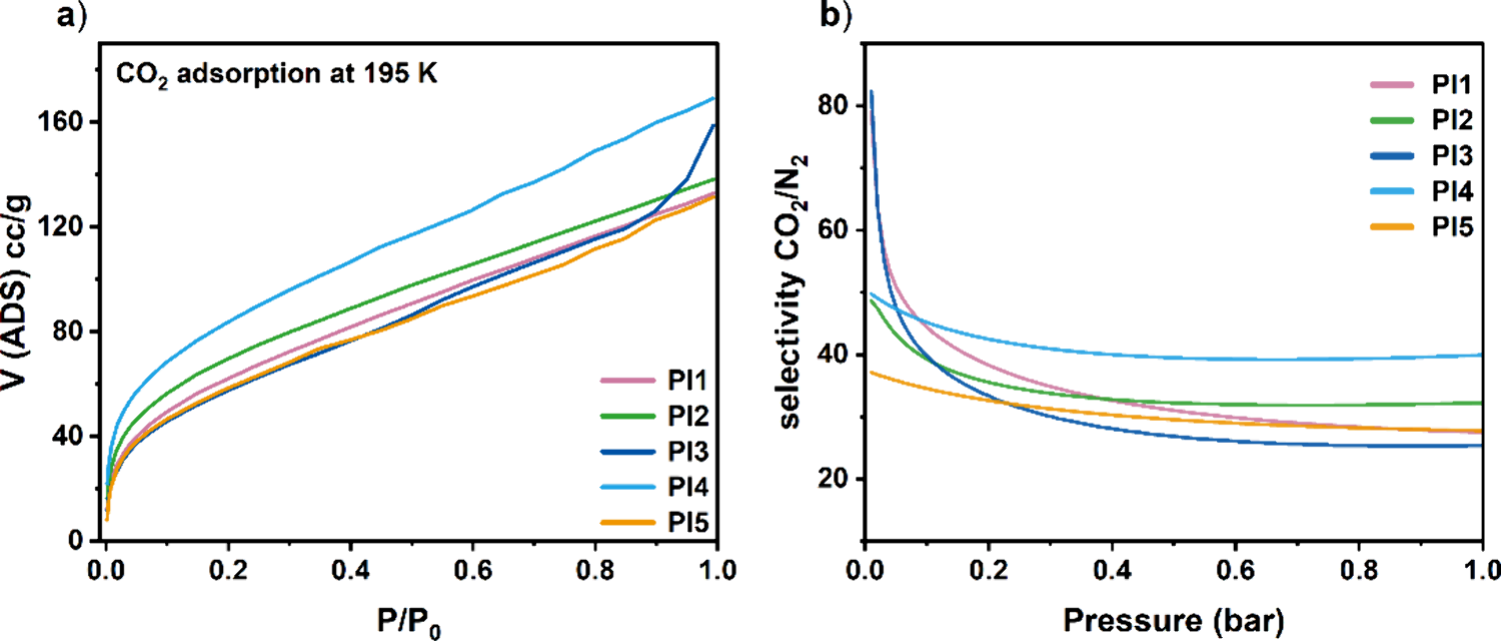
(a) CO_2_ adsorption isotherms measured at 195 K (desorption has been removed for clarity); (b) IAST selectivity for a hypothetical 15/85 CO_2_/N_2_ mixture of all PCP-PIs, calculated from CO_2_ and N_2_ adsorptions at 298 K.

Pore size distribution (PSD) analyses, calculated via NLDFT from CO_2_ adsorption at 273 K, show that all of the reported polyimides exhibit the characteristic pore structure of PIMs with distributions centered around 3.5, 5.2, and 8.2 Å. More specifically, the two PCP-PCP-based polyimides display nearly identical PSD profiles, which is consistent with their very similar overall porosity, Figure 6a. A similar observation can be made for the two 6FDA-based polymers, both of which exhibit almost indistinguishable porosity characteristics, Figure 6b. The most pronounced difference arises when comparing **PCP-PI4** and **PCP-PI3**: the former displays the highest porosity, whereas the latter has the lowest surface area, Figure 6c. We notice that **PCP-PI4** presents a more intense peak centered at approximately 3.5 Å, which corresponds to the region that defines ultramicroporosity.

**Figure 6:**
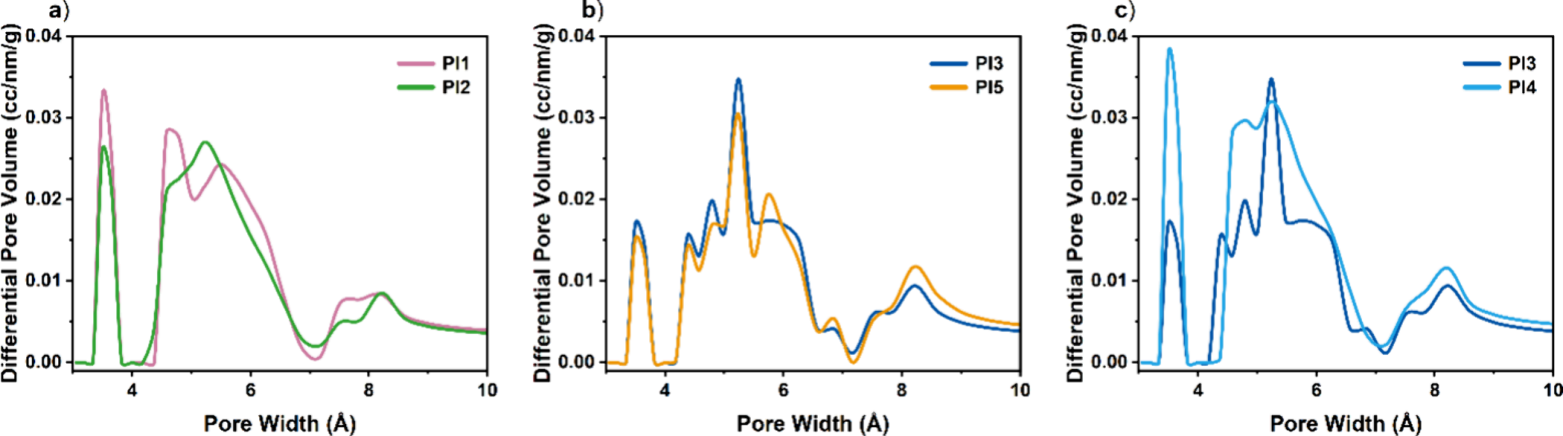
Series of pore size distributions, measured from CO_2_ at 273 K; (a) **PCP-PI1** and **PCP-PI2** (the two PCP-PCP PIs); (b) **PCP-PI3** and **PCP-PI5** (the two 6FDA PIs); and (c) **PCP-PI3** and **PCP-PI4** (6FDA vs EA-PI).

Scanning electron microscopy (SEM) revealed different morphologies and generally a heterogeneous distribution of particle sizes and shapes, including irregular grains and flakes between 5 and 250 μm of **PCP-PI1** and **PCP-PI4**, respectively, flaky material for **PCP-PI2**, 10–200 μm heterogeneous particles of **PCP-PI3**, and <30 μm grains of **PCP-PI5**, the smallest and most homogeneous particles from the analyzed polymers (Figures S27–S31). Energy-dispersive X-ray spectroscopy (EDX) analysis showed the expected homogeneous element distribution for all polymers (Figures S32–S36). Low content of N in the monomer unit resulted in low intensity signal compared to those of C, O, and F.

### 3.4 Thermal Stability

The thermogravimetric (TG) curves of the PCP-PIs are shown in Figure S21a, and the derivatives of these curves (DTG) are presented in Figure S21b. The mass loss between room temperature and 150 °C (maximum 2%) is attributed to the desorption of physisorbed water. Thermal decomposition of the polymers begins at temperatures between 385 and 440 °C (see Table S1). Polymers **PCP-PI2** and **PCP-PI3** show a small mass loss between 285–370 °C and 330–370 °C, respectively, while polymer **PCP-PI4** loses more than 4% of its mass in the range of 285–370 °C, which is easily attributed to the retro-Diels–Alder step that, at high temperatures, removes the ethylene bridge and confirms the successful insertion of the ethanoanthracene monomer.^39^ A mass loss of approximately 1% was observed for **PCP-PI5** in the temperature range of 160–255 °C and more than 4.5% between 300 and 435 °C.

### 3.5 Preparation of Films

All of the synthesized polyimides were evaluated for their solubility with particular attention to their potential application as membranes for gas separation. In this context, solubility plays a crucial role as only polymers that are fully soluble in a suitable solvent can be processed into robust and durable films. These membranes will be tested for gas permeability and selectivity, especially looking at commercially relevant combinations of gases, i.e., O_2_/N_2_, CO_2_/N_2_, and H_2_/N_2_.

In our study, not all of the prepared polyimides exhibited complete solubility in the tested organic solvents. Among the series, only **PCP-PI2** and **PCP-PI3** showed satisfactory solubility, both being partially soluble in chloroform and fully soluble in dimethylacetamide (DMAc) and **PCP-PI4** being partially soluble in chloroform. Although DMAc is not the ideal solvent for film casting due to its relatively high boiling point, which requires heating of the solution during film formation, it is still commonly employed in membrane fabrication.^6^ Films cast from such high-boiling, viscous solvents can nevertheless display excellent mechanical integrity and uniformity, provided the casting conditions are carefully controlled.^45^

A typical polyimide film was prepared by dissolving the polymer at a concentration of 3.5% (w/v) in an appropriate solvent and casting the resulting solution into a Petri dish. The solvent is then allowed to evaporate slowly under controlled conditions, leading to the formation of a thin, defect-free film. Depending on the amount of polymer used and the diameter of the Petri dish, the resulting films exhibited thicknesses in the range of approximately 50–120 μm.

Films obtained from **PCP-PI2** and **PCP-PI3** (Figure S1) showed limited mechanical robustness and tended to fracture upon bending. Nevertheless, they formed uniformly and adhered well to the casting surface, advantageous for subsequent gas permeability measurements.

## 4 Conclusion and Future Implications

In conclusion, we prepared a series of polyimides based on the PCP core containing interesting combinations of PCP dianilines and CP-bisanhydrides. The latter are very innovative, as it is very difficult to design and synthesize bisanhydrides, and most studies report changing of the dianilines instead. The series of PCP-based polyimides studied in this work demonstrate that the presence of the PCP core influences the packing of polymer chains in the solid state more than initially anticipated, resulting in a slightly lower microporosity than expected. Nevertheless, the polymers were found to exhibit good CO_2_ adsorption, reasonable solubility, and promising predicted CO_2_/N_2_ separation selectivity, particularly for the EA­(Me)-based polymer. These results indicate that, despite the somewhat denser packing, the innovative polymer design imparts characteristics that are favorable for carbon capture, storage, and other gas separation applications.

Although the polymers exhibited limited solubility, this aspect represents an opportunity for further optimization rather than a fundamental limitation. Future work will focus on improving solubility through structural modification such as the incorporation of suitable side chains (i.e., methyl or ethyl groups in selected monomers) as well as through a more systematic investigation of the polymerization conditions of dianiline monomers with paracyclophane bisanhydrides. Additionally, tailoring the comonomer ratio, particularly by increasing the fraction of monomers known to yield highly soluble polyimides, may offer an effective strategy to enhance processability while preserving the desirable material properties.

In summary, this study highlights the potential of PCP-based polyimides as a versatile platform for designing microporous polymers with tunable adsorption and separation properties, suggesting that further structural optimization could enhance their performance even further.

## Supplementary Material



## Notes

Experimental instrumentation; materials and methods; additional experimental details and characterization data for monomers and polymers, including ^1^H NMR, ^13^C NMR, mass spectrometry, IR spectroscopy, and thermogravimetric analysis; gas adsorption isotherms measured at different temperatures and pore size distribution analysis; additional polymer characterization data, including WAXD, SEM, and EDX (PDF)
