# Supplementary material for: [2.2]Paracyclophane-Based Polyimides of Intrinsic Microporosity for Gas Separation
Source: ACS Appl Polym Mater. 2026 Mar 31;8(7):5248–57. doi: 10.1021/acsapm.6c00411 (PMC13077630; doi:10.1021/acsapm.6c00411)
Supplement: Supplementary file 1 [file ap6c00411_si_001.pdf]

Supporting Information

[2.2]Paracyclophane-Based Polyimides of Intrinsic Microporosity for Gas Separation

Yuting Li,[1] C. Grazia Bezzu,[2] Anže Zupanc,[3] Luis Simbari,[3] Shrestha Banerjee,[3] Dominik J. Kubicki, [3] Tomislav Friščić,[3] Marjan Jereb,[4] Ross D. Jansen-van Vuuren,[4] Mariolino Carta,[2*] Stefan Bräse[1*]*

[1]Institute of Organic Chemistry (IOC) Karlsruhe Institute of Technology (KIT), Kaiserstraße 12, 76131 Karlsruhe, Germany

[2]Department of Chemistry Faculty of Science and Engineering Swansea University Swansea SA2 8PP, UK

[3]School of Chemistry, University of Birmingham, Edgbaston, Birmingham B15 2TT, UK

[4]Faculty of Chemistry and Chemical Technology, University of Ljubljana, Večna pot 113, 1000 Ljubljana, Slovenia

[5]Instituto de Síntesis Química y Catálisis Homogénea, CSIC-Universidad de Zaragoza, C/Pedro Cerbuna 12, Facultad de Ciencias, Zaragoza 50009, Spain

Table of Contents

1 General Remarks 3

2 Synthesis Procedures 9

2.1 Synthesis of the monomer 9

2.2 Synthesis of the polymer 14

3 Qualitative Analysis 18

3.1 MALDI-TOF analysis 18

3.2 Solid-state NMR spectra of all PCP-PIs 19

3.2.1 Solid-state NMR experimental parameters 19

3.2.2 NMR spectra of **PCP-PI1** 20

3.2.3 NMR spectra of **PCP-PI2** 22

3.2.4 NMR spectra of **PCP-PI3** 24

3.2.5 NMR spectra of **PCP-PI4** 26

3.2.6 NMR spectra of **PCP-PI5** 27

3.3 FT-IR Spectra of Polymers 30

3.4 TG and DTG analysis 31

4 Porosity and Selectivity Measurements of PCP-PIs 33

5 Wide-angle X-ray diffraction (WAXD) analyses 36

6 SEM Images and EDX mapping of the Polymers 37

6.1 SEM images 37

6.2 EDX mapping 39

7 References 44

1 General Remarks

Materials and Methods

The catalysts, starting materials, solvents, and reagents were purchased from ABCR, ACROS, ALFA AESAR, APOLLO SCIENTIFIC, CARBOLUTION, CHEMPUR, FLUKA, FLUOROCHEM, MERCK, RIEDEL-DE HAËN, SIGMA ALDRICH, STREM, TCI, or THERMO FISHER SCIENTIFIC and used without further purification unless stated otherwise.

Solvents of technical quality were purified by distillation or with the solvent purification system MB SPS5 (acetonitrile, dichloromethane, diethyl ether, tetrahydrofuran, toluene) from MBRAUN. Solvents of *p.a.* quality were purchased from ACROS, FISHER SCIENTIFIC, SIGMA ALDRICH, Roth, or RIEDEL-DE HAËN and were used without further purification. Diphenyl ether and α,α,α,-trifluoro toluene were distilled over calcium hydride. *n*-Pentane was distilled over sodium and benzophenone. Other solvents were obtained from commercial suppliers: anhydrous benzene (SIGMA ALDRICH, <0.005% water), anhydrous *N*,*N*-dimethylformamide (SIGMA ALDRICH, <0.005% water), anhydrous 1,4-dioxane (SIGMA ALDRICH, <0.005% water), anhydrous dimethyl sulfoxide (SIGMA ALDRICH, <0.005% water), anhydrous ethanol (SIGMA ALDRICH, <0.005% water), anhydrous methanol (SIGMA ALDRICH, <0.005% water), anhydrous isopropanol (SIGMA ALDRICH, <0.005% water).

Abbreviations: cHex = cyclohexane, EtOAc = ethyl acetate, DCM = methylene chloride, M.p.= melting point

Oxygen-free solvents were obtained by freeze-pump-thaw (three cycles) technique.

Air- and moisture-sensitive reactions were carried out under an argon atmosphere in oven-dried glassware using standard Schlenk techniques.

For certain reactions, flat-bottom crimp neck vials from CHROMAGLOBE with aluminum crimp caps were used.

Liquids were added with a stainless-steel cannula, and solids were added in powdered form.

Reactions at low temperatures were cooled using flat dewars produced by ISOTHERM (Karlsruhe) with water/ice or isopropanol/dry ice mixtures.

Solvents were evaporated under reduced pressure at 45/40 °C using a rotary evaporator. For solvent mixtures, each solvent was measured volumetrically.

Flash column chromatography was performed using MERCK silica 60 (0.040 × 0.063 mm, 230–400 mesh ASTM) and quartz sand (glowed and purified with hydrochloric acid).

Low-temperature N_2 (77 K) and CO_2 (195 K, 273 K and 298 K) adsorption/desorption measurements of polymer powders were made using an Anton Paar Nova600. Samples were degassed for 800 min at 80 °C under high vacuum prior to analysis.

The data were analysed with the software provided with the instrument. NLDFT analyses were performed to calculate the pore size distribution and volume, considering a carbon equilibrium transition kernel at 273 K based on a slit-pore model; the kernel is based on a common, one centre, Lennard-Jones model.

Reaction Monitoring

All reactions were monitored by thin-layer chromatography (TLC) using silica-coated aluminum plates (MERCK, silica 60, F254). UV active compounds were detected with a UV-lamp at 254 nm and 366 nm excitation.

When required, vanillin (15 g in 250 mL ethanol and 2.5 mL conc. H_2SO_4), ninhydrin (1.5 g in 100 mL ethanol and 3.0 mL acetic acid), Seebach solution (2.5 g phosphomolybdic acid, 1.0 g $Ce(SO_4)_2$ in 94 mL water and 6.0 mL conc. H_2SO_4), molybdato phosphate (5% phosphor molybdic acid in ethanol), potassium permanganate (0.45 g potassium permanganate and 2.35 g of sodium carbonate in 90 ml of water), 2,4-DNP (1.2 g 2,4-dinitrophenylhydrazin in 8.0 mL water, 20 mL ethanol and 6.0 mL conc. H_2SO_4) or bromocresol green (40 mg in 100 mL ethanol, 0.1 M sodium hydroxide in water was added until the solution turned blue) were used as TLC-stain.

GC-MS (gas chromatography-mass spectrometry) measurements were performed on an AGILENT TECHNOLOGIES model 6890N (electron impact ionization), equipped with an AGILENT 19091S-433 column (5% phenyl methyl siloxane, 30 m, 0.25 µm) and a 5975B VL MSD detector with a turbopump. Helium was used as a carrier gas.

Nuclear Magnetic Resonance Spectroscopy (NMR)

NMR spectra of monomer were recorded on a BRUKER Avance 400 NMR instrument at 400 MHz for ^{1}H NMR, 101 MHz for ^{13}C NMR. The ^{1}H NMR spectra of the polyimide were recorded in the solvent stated using an Avance Bruker DPX 500 (500 MHz) instruments, at 500 MHz for ^{1}H NMR, 126 MHz for ^{13}C NMR. Chemical shifts are reported in ppm relative

to $(CH_3)_4Si$ (TMS) using the CH_3 signal of L-alanine (δ = 20.5 ppm) as a secondary solid reference,

The NMR spectra were recorded at room temperature in deuterated solvents acquired from EURISOTOP, SIGMA ALDRICH, or DEUTERO. The chemical shift δ is displayed in parts per million [ppm] and the references used were the ^{1}H and ^{13}C peaks of the solvents themselves:

d_1-chloroform ($CDCl_3$): 7.26 ppm for ^{1}H and 77.16 ppm for ^{13}C

For the characterization of centrosymmetric signals, the signal's median point is given, for multiplets the signal range. The following abbreviations are used to describe the proton splitting pattern: d = doublet, t = triplet, m = multiplet, dd = doublet of doublet, ddd = doublet of doublet of doublet, dddd = doublet of doublet of doublet of doublet, dt = doublet of triplet. Absolute values of the coupling constants "*J*" are given in Hertz [Hz] and decreasing order. Signals of the ^{13}C spectrum are assigned by distortionless enhancement by polarization transfer (DEPT) spectra DEPT90 and DEPT135 or phase edited heteronuclear single quantum coherence (HSQC).

Solid State Nuclear Magnetic Resonance Spectroscopy

Solid-state ^{1}H (400.6 MHz), ^{19}F (376.4 MHz), ^{13}C (100.6 MHz) MAS NMR spectra were recorded on a Bruker Avance Neo 9.4 T spectrometer equipped with a 3.2 mm HX MAS probe; ^{15}N (40.59 MHz) MAS NMR spectra were recorded using a 4 mm HX MAS probe. The powder samples were packed into 3.2 mm or 4 mm zirconia rotors, which were spun at 15 kHz (3.2 mm) or 8 kHz (4 mm) using dry nitrogen. ^{13}C chemical shifts were referenced using solid adamantane at 38.38 ppm (for CH_2) as a secondary reference, corresponding to 0 ppm in TMS. ^{1}H, ^{19}F and ^{15}N chemical shifts were referenced in accordance with the IUPAC recommendation (ratio of gyromagnetic ratios). For ^{1}H and ^{19}F NMR a 100 kHz RF field amplitude was used, and spectra were acquired using a Hahn echo (90°-180°) sequence with an inter-pulse delay equal to one rotor period. For ^{1}H-^{13}C CP measurements, a 62 kHz ^{13}C spin-lock RF field amplitude and a 74 kHz ^{1}H spin-lock RF field amplitude (ramped from 80% to 100% for ^{1}H) were used with a 1 ms contact time and 80 kHz ^{1}H decoupling. For ^{1}H-^{15}N CP measurements, a 55 kHz ^{15}N spin-lock RF field amplitude and a 40 kHz ^{1}H spin-lock RF field amplitude (ramped from 80% to 100% for ^{1}H) were used with a 7 ms contact time and 50 kHz ^{1}H decoupling. Longitudinal relaxation times T_1 were measured using a saturation-recovery sequence. Further experimental parameters and measured T_1 values are given in Table S1.

Infrared Spectroscopy (IR)

The infrared spectra were recorded with a BRUKER, Alpha P instrument. All samples were measured by attenuated total reflection (ATR). The positions of the absorption bands are given in wavenumbers $\tilde{\nu}$ in cm^{-1} and were measured in the range from 3600 cm^{-1} to 500 cm^{-1}.

Characterization of the absorption bands was done in dependence of the absorption strength with the following abbreviations: vs (very strong, 0−9%), s (strong, 10−39%), m (medium, 40−69%), w (weak, 70−89%), vw (very weak, 90−100%).

Mass Spectrometry (MS)

Electron ionization (EI) and fast atom bombardment (FAB) experiments were conducted using a FINNIGAN, MAT 90 (70 eV) instrument, with 3-nitrobenzyl alcohol (3-NBA) as matrix and reference for high resolution. For the interpretation of the spectra, molecular peaks $[M]^+$ or peaks of protonated molecules $[M+H]^+$ and characteristic fragment peaks are indicated with their mass-to-charge ratio (*m/z*) and their intensity in percent, relative to the base peak (100%). In the case of high-resolution measurements, the maximum tolerated error is ±5 ppm.

APCI (atmospheric pressure chemical ionization) and ESI (electrospray ionization) experiments were recorded on a Q-Exactive (Orbitrap) mass spectrometer (THERMO FISHER SCIENTIFIC, San Jose, CA, USA) equipped with a HESI II probe to record high resolution. The tolerated error is ±5 ppm of the molecular mass. The spectra were interpreted by molecular peaks $[M]^+$or peaks of protonated molecules $[M+H]^+$ and characteristic fragment peaks and indicated with their mass-to-charge ratio (*m/z*) and intensity in percent, relative to the base peak (100%).

Matrix-assisted laser desorption/ionization time-of-flight analysis (MALDI-TOF)

The MALDI-TOF analysis was performed using Mass Spectrometer Bruker ultrafleXtreme MALDI TOF/TOF instrument. Stains were prepared on MTP ground stainless steel target (DE1580TA) with 384 wells. Matrix used was *trans*-2-[3-(4-*tert*-butylphenyl)-2-methyl-2-propenylidene]malononitrile (DCTB) dissolved in chloroform at a concentration of 10 mg/ml and sample solutions prepared chloroform had a target concentration of 5 mg/ml, although it was difficult to determine complete dissolution. The solutions were mixed under ultrasound for 3 minutes, after which a 2 µL quantity was added portion-wise onto a well for staining, taking care there is no spillage or cross-contamination. The spectra were recorded in the range 400–5000 m/z.

Melting point

Melting points were detected on an OptiMelt MPA100 device from STANFORD RESEARCH SYSTEM.

Thermogravimetric analysis (TGA)

Thermogravimetric (TG) measurements were performed using a Mettler Toledo TGA/DSC1 instrument in a temperature range from 25 to 600 °C with a heating rate of 5 K/min. During measurement, the furnace was purged with nitrogen at a flow rate of 50 mL/min. Samples with an initial mass of approximately 5 mg were placed in 150 µL platinum crucibles. To ensure an inert atmosphere during heating, the furnace with the inserted sample was purged with nitrogen (50 mL/min) at room temperature for 10 min. In all measurements, the blank curve was subtracted.

IAST Selectivity calculation

The ideal adsorption solution theory (IAST) of Myers and Prausnitz [1] is typically used to the selectivity of binary mixtures of gases from the single isotherms. The isotherms were fitted with Dual-Site Langmuir-Freundlich using the software IAST++ [2] and the selectivity (S) was calculated according to the formula:

$$S = \frac{Q_{CO2}}{Q_{N2}} \times \frac{P_{N2}}{P_{CO2}}$$

where:

- P_{CO2} is the partial pressure of CO_2
- P_{N2} is the partial pressure of N_2
- Q_{N2} is the N_2 uptake
- Q_{CO2} is theCO_2 uptake

Powder X-ray diffraction (PXRD)

PXRD data was collected using a Malvern Panalytical Aeris diffractometer operating in reflection mode, with a CuK_α source (λ=1.5406 Å) with a nickel filter. In a typical experiment, around 30 mg of a sample were thinly and uniformly distributed across a silicon wafer, and data recorded in the 2θ-range 5–60 °, with a step size of 0.02173288 °.

Scanning electron microscopy and energy-dispersive X-ray spectroscopy (SEM-EDX)

A HITACHI TM4000plus benchtop scanning electron microscope (SEM) with AztecOne EDX analyser was utilised to examine the microstructures and element distribution of polymers. The sample powders were placed onto a carbon tape and analysed at 15 kV in backscattered electron mode.

2 Synthesis Procedures

2.1 Synthesis of the monomer

Dimethyl 4-(4,4,5,5-tetramethyl-1,3,2-dioxaborolan-2-yl)benzene-1,2-dicarboxylate

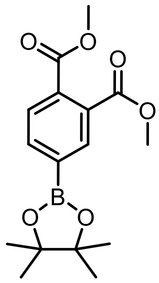

In a sealable vial, dimethyl 4-bromobenzene-1,2-dicarboxylate (2.00 g, 7.32 mmol, 1.00 equiv), bis(pinacolato)diboron (2.23 g, 8.79 mmol, 1.20 equiv), Pd(dppf)Cl_2 (268 mg, 366 µmol, 0.0500 equiv), and potassium acetate (2.16 g, 22.0 mmol, 3.00 equiv) were dissolved under argon atmosphere in 32 mL of dry dioxane. The mixture was heated to 85 °C for 24 h and then cooled to 22 °C. The mixture was filtered through Celite, and the mixture was extracted with EtOAc (3*30 mL). The combined organic layer was washed with brine (3 x 30 mL). After drying over $NaSO_4$, the solvent was removed under reduced pressure. The crude solid was purified by flash column chromatography (silica, cHex/EtOAc 3:1) to obtain the title product dimethyl 4-(4,4,5,5-tetramethyl-1,3,2-dioxaborolan-2-yl)benzene-1,2-dicarboxylate (2.30 g, 7.18 mmol, 98% yield) as a colorless oil.

M.p.: 95 °C.

R_f = 0.5 (cyclohexane/ethyl acetate 3:1).

^{1}H NMR (400 MHz, $CDCl_3$ [7.26 ppm], ppm), δ = 8.15 (d, J = 1.2 Hz, 1H, H_{Ar}), 7.94 (dd, J = 7.6, 1.2 Hz, 1H, H_{Ar}), 7.69 (d, J = 7.6 Hz, 1H, H_{Ar}), 3.90 (s, 3H, CH_3), 3.89 (s, 3H, CH_3), 1.34 (s, 12H, CH_3) Impurities: spectrum contains EtOAc signal at 4.12 ppm, 2.05 ppm and 1.26 ppm.

^{13}C NMR (101 MHz, $CDCl_3$ [77.16 ppm], ppm), δ = 168.2 (CO, 1C), 168.0 (CO, 1C), 137.4 (CH, 1C, C_{Ar}), 135.1 (CH, 1C, C_{Ar}), 134.2 (C_q, 1C, C_{Ar}), 131.0 (C_q, 1C, C_{Ar}), 128.0 (CH, 1C, C_{Ar}), 84.4 (C_q, 2C, C_{Ar}), 52.7 (CH_3, 1C), 52.6 (CH_3, 1C), 25.0 (CH_3,4C). Missing signals: a signal (1C, C_q) is missing due to the coupling with boron.

^{11}B NMR (128 MHz, Chloroform-d [77.16 ppm], ppm) δ = 30.5.

MS (ESI+, DCM): 321 (100) $[M+H]^+$, 289 (54) $[M-OCH_3]^+$.

HRMS (ESI+): calcd. for $C_{16}H_{21}BO_6$ $[M+H]^+$: 321.1504, found 321.1507.

IR (ATR, ṽ) = 2979 (w), 2952 (w), 1727 (vs), 1611 (w), 1561 (w), 1497 (w), 1435 (m), 1414 (w), 1392 (s), 1381 (m), 1360 (vs), 1329 (s), 1282 (vs), 1259 (vs), 1214 (m), 1191 (m), 1166 (m), 1142 (vs), 1125 (vs), 1101 (vs), 1069 (vs), 1004 (w), 976 (s), 962 (s), 926 (w), 880 (m),

850 (vs), 819 (m), 803 (m), 793 (m), 769 (m), 711 (s), 669 (s), 629 (w), 601 (w), 577 (w), 530 (w), 520 (w), 510 (w), 499 (w), 493 (w), 456 (w), 448 (w), 429 (w), 424 (w), 418 (w), 405 (w), 395 (w), 377 (m) cm^{-1}.

Additional information on the chemical synthesis is available via Chemotion repository:

https://doi.org/10.14272/reaction/SA-FUHFF-UHFFFADPSC-RLDRAXBPIR-UHFFFADPSC-NUHFF-NUHFF-NUHFF-ZZZ

Additional information on the analysis of the target compound is available via Chemotion repository: https://doi.org/10.14272/RLDRAXBPIRSRSV-UHFFFAOYSA-N.1

Tetramethyl 4,4'-(1,4(1,4)-dibenzenacyclohexaphane-12,42-diyl)diphthalate

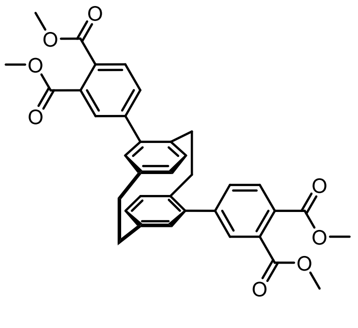

In a sealable vial, 4,15-dibromo[2.2]paracyclophane (500 mg, 1.37 mmol, 1.00 equiv), dimethyl 4-(4,4,5,5-tetramethyl-1,3,2-dioxaborolan-2-yl)benzene-1,2-dicarboxylate (1.09 g, 3.41 mmol, 2.50 equiv), $Pd(PPh_3)_4$ (78.9 mg, 68.3 µmol, 0.0500 equiv) and potassium phosphate (562 mg, 4.10 mmol, 3.00 equiv) were dissolved under argon atmosphere in 20 mL of dioxane and 5 mL of H_2O. The mixture was heated to 90 °C for 16 h and then cooled to 22 °C. The mixture was extracted with DCM, and the solvent was removed under reduced pressure. The crude solid was purified by flash column chromatography (silica, cHex/EtOAc 10:1) to obtain the title product tetramethyl 4,4'-(1,4(1,4)-dibenzenacyclohexaphane-12,42-diyl)diphthalate (610 mg, 91% purity, 937 µmol, 69% yield) as an off-white solid.

M.p.: 178 °C.

R_f = 0.34 (cyclohexane/ethyl acetate 3:1).

^{1}H NMR (400 MHz, $CDCl_3$ [7.26 ppm], ppm) δ = 7.86 (d, J = 8.0 Hz, 2H, H_{Ar}), 7.81 (d, J = 1.5 Hz, 2H, H_{Ar}), 7.63 (dd, J = 8.0, 1.8 Hz, 2H, H_{Ar}), 6.73–6.72 (m, 2H, H_{Ar}), 6.63–6.62 (m, 4H, H_{Ar}), 3.97 (s, 6H, CH_3), 3.96 (s, 6H, CH_3), 3.30–3.19 (m, 2H, H_{Pc}), 3.19–3.05 (m, 4H, H_{Pc}), 2.61–2.47 (m, 2H, H_{Pc}) Impurities: spectrum contains water signal at 1.57 ppm and other impurities throughout the spectrum. The yield was adjusted accordingly.

^{13}C NMR (101 MHz, $CDCl_3$ [77.16 ppm], ppm) δ = 168.3 (CO, 2C), 167.9 (CO, 2C), 144.1 (C_q, 2C, C_{Ar}), 140.6 (C_q, 2C, C_{Ar}), 140.1 (C_q, 2C, C_{Ar}), 137.4 (C_q, 2C, C_{Ar}), 132.8 (C_q, 2C, C_{Ar}), 132.5 (CH, 2C, C_{Ar}), 132.2 (CH, 2C, C_{Ar}), 132.0 (CH, 2C, C_{Ar}), 131.9 (CH, 2C, C_{Ar}), 129.9

(C_q, 2C, C_{Ar}), 129.7 (CH, 2C, C_{Ar}), 129.5 (CH, 2C, C_{Ar}), 52.9 (CH_3, 2C), 52.7 (CH_3, 2C), 35.0 (CH_2, 2C, C_{Pc}), 33.3 (CH_2, 2C, C_{Pc}).

MS (ESI+, DCM): 593 (4) $[M+H]^+$, 568 (5), 552 (10), 551 (8), 550 (14). Unknown adducts: 639 (27), 605 (100).

HRMS (ESI+): calcd. for $C_{36}H_{33}O_8$ $[M+H]^+$: 593.2170, found 593.2182.

IR (ATR, ṽ) = 2986 (vw), 2972 (w), 2946 (w), 2929 (w), 2894 (w), 2851 (w), 1717 (vs), 1599 (m), 1564 (w), 1482 (vw), 1453 (w), 1432 (s), 1388 (w), 1276 (vs), 1252 (vs), 1190 (m), 1170 (w), 1125 (vs), 1071 (vs), 960 (m), 929 (w), 911 (w), 894 (w), 873 (w), 853 (m), 830 (w), 820 (m), 785 (m), 772 (s), 730 (m), 710 (m), 676 (m), 653 (w), 647 (w), 637 (w), 596 (w), 562 (w), 550 (w), 540 (w), 524 (w), 507 (w), 483 (w), 473 (w), 449 (w), 425 (w), 411 (w), 395 (w), 377 (w) cm^{-1}.

Additional information on the chemical synthesis is available via Chemotion repository:

https://doi.org/10.14272/reaction/SA-FUHFF-UHFFFADPSC-VJNUYUKQJF-UHFFFADPSC-NUHFF-NUHFF-NUHFF-ZZZ

Additional information on the analysis of the target compound is available via Chemotion repository: https://doi.org/10.14272/VJNUYUKQJFYKQS-UHFFFAOYSA-N.1

4,4'-(1,4(1,4)-Dibenzenacyclohexaphane-12,42-diyl)diphthalic acid

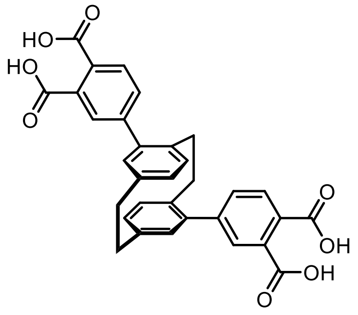

To a 100 mL round-bottom flask equipped with a magnetic bar and a refluxing condenser, tetramethyl 4,4'-(1,4(1,4)-dibenzenacyclohexaphane-12,42-diyl)diphthalate (500 mg, 844 µmol, 1.00 equiv) and KOH (1.89 g, 33.7 mmol, 40.0 equiv) was added to THF (16.0 mL) and water (24.0 mL). The mixture was stirred at 80 °C in an oil bath for 22 h. After cooling to 25 °C, the organic layer was evaporated, and the remaining aqueous phase was acidified by hydrochloric acid (2 M) to pH = 5. The precipitate was filtered off and dried under high vacuum to afford the title product 4,4'-(1,4(1,4)-dibenzenacyclohexaphane-12,42-diyl)diphthalic acid (423 mg, 788 µmol, 93% yield) as a colorless solid.

M.p.: 179 °C.

^1H NMR (400 MHz, DMSO [2.05 ppm], ppm) δ = 13.41 (s, 4H, COOH), 8.10–7.41 (m, 6H, H_{Ar}), 6.98–6.31 (m, 6H, H_{Ar}), 3.71–2.71 (m, 8H, H_{pc}).

^{13}C NMR (101 MHz, DMSO-d6 [39.52 ppm] ppm) δ = 169.2 (CO, 2C), 168.9(CO, 2C), 143.3 (C_q, 2C, C_{Ar}), 140.9 (C_q, 2C, C_{Ar}), 140.6 (C_q, 2C, C_{Ar}), 137.2 (C_q, 2C, C_{Ar}), 134.4 (C_q, 2C, C_{Ar}), 132.6 (CH, 4C, C_{Ar}), 132.2 (CH, 2C, C_{Ar}), 131.8 (CH, 2C, C_{Ar}), 131.5 (C_q, 2C, C_{Ar}), 130.3–129.8 (m, CH, 4C, C_{Ar}), 34.7 (CH_2, 2C, C_{Pc}), 33.3 (CH_2, 2C, C_{Pc}).

MS (ESI-, DCM), m/z (%): 535 (39) $[M-H]^-$, 268 (34), 267 (100) $[C_{16}H_{11}O_4]^-$.

HRMS (ESI-): calcd. for $C_{32}H_{24}O_4$ $[M]^-$: 535.1398, found 535.1401.

IR (ATR, ṽ) = 3233 (w), 2476 (w) (COOH), 1690 (vs), 1598 (vs), 1562 (s), 1394 (w), 1390 (w), 1371 (m), 1228 (vs), 1170 (m), 1135 (s), 1068 (s), 792 (m), 766 (w) cm^{-1}.

Additional information on the chemical synthesis is available via Chemotion repository:

https://doi.org/10.14272/reaction/SA-FUHFF-UHFFFADPSC-SGYKYHRHGH-UHFFFADPSC-NUHFF-NUHFF-NUHFF-ZZZ

Additional information on the analysis of the target compound is available via Chemotion repository: https://doi.org/10.14272/SGYKYHRHGHIMLU-UHFFFAOYSA-N.1

5,5'-((4,15-[2.2]Paracyclophandiyl)bis(isobenzofuran-1,3-dione) (PCP-BA)

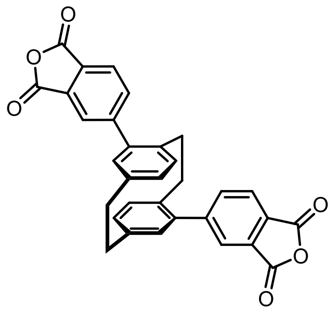

4,4'-(1,4(1,4)-Dibenzenacyclohexaphane-12,42-diyl)diphthalic acid (1.20 g, 2.24 mmol, 1.00 equiv) was added to acetic anhydride (11.4 g, 10.6 mL, 112 mmol, 50.0 equiv). The mixture was refluxed for 3 h. The excess acetic anhydride was evaporated under vacuum, and then the crude product was washed with DCM (20 mL) and acetone (20 mL). The off-white solid was dried under vacuum to yield the product 4,15-bis(5,5'(isobenzofuran-1,3-dione))[2.2]paracyclophane (750 mg, 1.50 mmol, 67% yield) as a pale green solid.

M.p.: >350 °C.

IR (ATR, ṽ) = 1846 (m), 1769 (vs), 1608 (w), 1332 (w), 1292 (w), 1272 (w), 1256 (w), 1239 (m), 1106 (w), 933 (w), 890 (vs), 851 (w), 829 (w), 742 (m), 727 (w), 715 (w), 693 (w), 681 (w), 654 (w), 581 (w), 571 (w), 548 (w), 480 (w), 418 (w), 388 (w) cm^{-1}.

MS (ASAP-): 500 (67) $[M]^-$, 452 (49), 308 (100), 278 (61), 113 (20). Spectra contains starting material at 535 (21).

HRMS (ASAP-): calcd. for $C_{32}H_{20}O_6$ $[M]^-$: 500.1265, found 500.1257.

Additional information on the chemical synthesis is available via Chemotion repository:

https://doi.org/10.14272/reaction/SA-FUHFF-UHFFFADPSC-OEDVNYZBGR-UHFFFADPSC-NUHFF-NUHFF-NUHFF-ZZZ

Additional information on the analysis of the target compound is available via Chemotion repository: https://doi.org/10.14272/OEDVNYZBGRXWAA-UHFFFAOYSA-N.1

2.2 Synthesis of the polymer

General procedure A.

The bis-anhydride was dissolved in ethanol in a two-necked flask equipped with Dean-Stark apparatus and reflux condenser, under a nitrogen atmosphere. Triethylamine was added and the mixture was refluxed for 1 h. The side arm was opened to remove the solvent under a stream of nitrogen to give a highly viscous solution.

The Dean Stark trap was filled with toluene before half the amount of solvent, NMP:toluene (4:1 mixture), and the diamine were added, followed by the other aliquot solvent. The reaction mixture was heated at 80 °C for 1 h and then the temperature gradually raised to 200 °C. The reaction was maintained at this temperature until the desired viscosity was achieved. The mixture was cooled to room temperature and diluted with chloroform. The mixture was poured into ethanol to precipitate a solid. The solid was collected by filtration, washed with ethanol until the washings were clear. Chloroform was added to the resulting solid followed by methanol and the resulting gel/solid was filtered off. The latter treatment was repeated, then the powder was refluxed in methanol for 24 h, filtered and then dried in a vacuum oven at 100 °C for 8 h to afford the desired polymer.

PCP-PI1 from pseudo-*meta*-PCP-bisanhydride (PCP-BA) and pseudo-*para*-PCP-dianiline (PCP-*p*-DA)

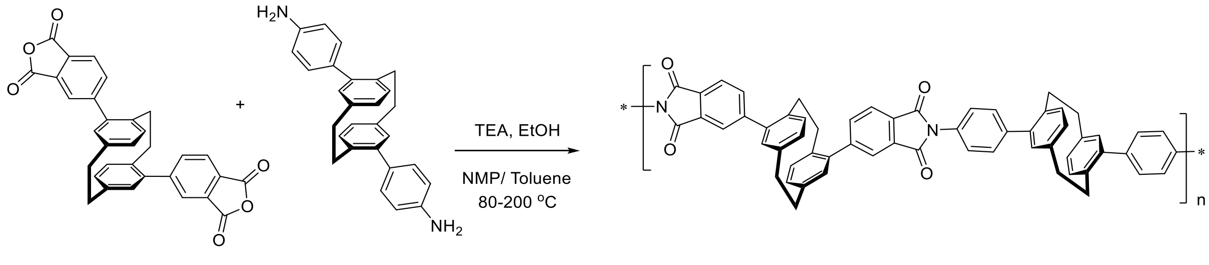

General procedure A was followed using PCP-*p*-DA (0.234 g, 0.60 mmol), PCP-BA (0.300 g, 0.60 mmol), ethanol (10 mL), triethylamine (0.303 g, 0.418 mL, 3.00 mmol), and NMP:toluene (5 mL) to afford PCP-PI1 (0.470 g, 92%) as a dark brown insoluble powder.

ν_{max} (cm^{-1}): 2930, 1773, 1712, 1364, 818.

BET surface area (CO_2, 195K) = 302 m^2/g; total pore volume = 0.2814 cm^3/g at (P/Po = 0.98).

PCP-PI2 from pseudo-*meta*-PCP-bisanhydride (PCP-BA) and pseudo-*meta*-PCP-dianiline (PCP-*m*-DA)

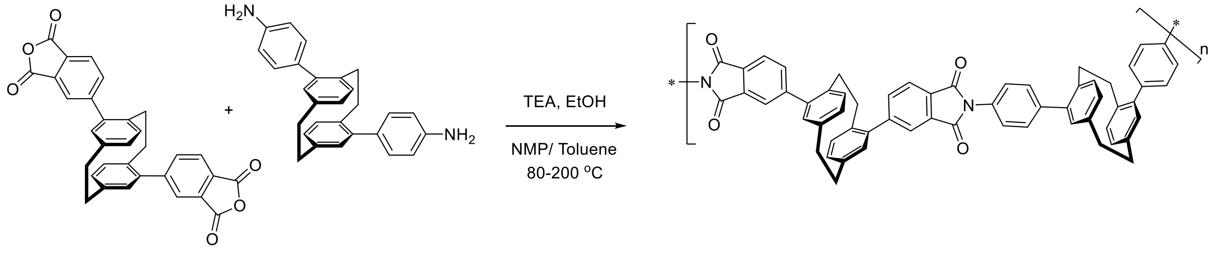

General procedure A was followed using PCP-*m*-DA (0.234 g, 0.60 mmol), PCP-BA (0.300 g, 0.60 mmol), ethanol (10 mL), triethylamine (0.303 g, 0.418 mL, 3.0 mmol), and NMP:toluene (4:1) (5 mL) to afford PCP-PI2 (0.440 g, 86 %) as a light brown powder.

ν_{max} (cm^{-1}): 3035, 2853, 1775, 1714, 1513, 1360, 833.

^1H NMR (500 MHz, CDCl3): δ 8.17 – 8.06 (br m, 4H, ArH), 7.92 (br d, 2H, *J* = 8.19 Hz, ArH), 7.70 – 7.60 (br m, 8H, ArH), 6.86 (br s, 2H, ArH), 6.83 – 6.66 (br m, 8H, ArH), 6.63 (br d, 2H, *J* = 7.81 Hz, ArH), 3.41 – 3.06 (br m, 12H, CH2), 2.80 – 2.63 (br m, 4H, CH2).

BET surface area (CO_2, 195K) = 335 m^2/g; total pore volume 0.2925 cm^3/g at (P/Po = 0.98).

PCP-PI3 from 6-FDA and pseudo-*meta*-PCP-dianiline (PCP-*m*-DA)

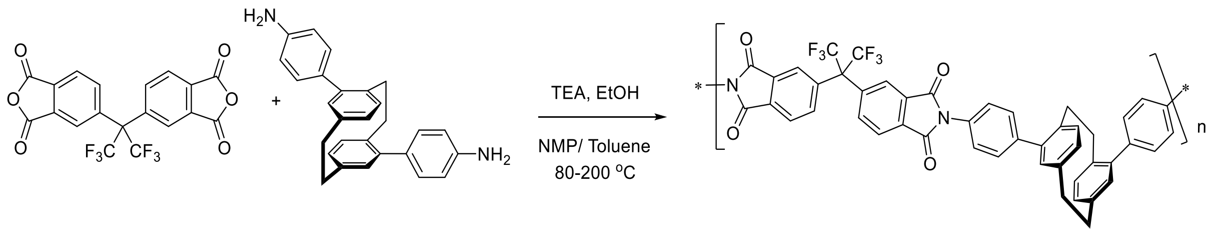

General procedure A was followed using PCP-*m*-DA (0.352 g, 0.90 mmol), 4,4-(hexafluoroisopropylidene)-diphthalic anhydride (6-FDA) (0.400 g, 0.90 mmol), ethanol (10 mL), triethylamine (0.455 g, 0.630 mL, 4.5 mmol), and NMP:toluene (4:1) (5 mL) to afford PCP-PI3 (0.510 g, 72 %) as a pale brown powder.

ν_{max} (cm^{-1}): 2930, 1785, 1721, 1370, 1256, 1209, 1192, 837, 720.

^1H NMR (500 MHz, CDCl3): δ 8.14 – 8.04 (br m, 2H, ArH), 8.00 (br s, 2H, ArH), 7.93 (br s, 2H, ArH), 7.69 – 7.60 (br m, 4H, ArH), 7.56 (br d, 4H, *J* = 7.90 Hz, ArH), 6.75 (br s, 2H, ArH), 6.72 (br d, 2H, *J* = 8.21 Hz, ArH), 6.59 (br d, 2H, *J* = 7.81 Hz, ArH), 3.22 (br s, 4H, CH2), 3.17– 3.05 (br m, 2H, CH2), 2.71 (br s, 2H, CH2).

BET surface area (CO_2, 195K) = 281 m^2/g; total pore volume 0.2932 cm^3/g at (P/Po = 0.98).

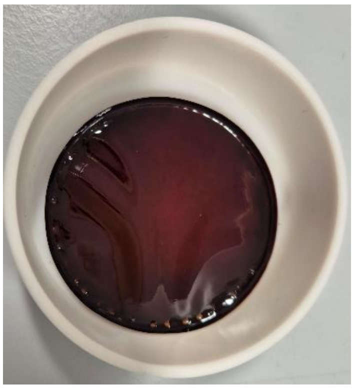

Figure S1. Film of PCP-PI3 cast from DMAc at 50 °C.

PCP-PI4 from pseudo-*meta*-PCP-bisanhydride (PCP-BA) and EA(Me)

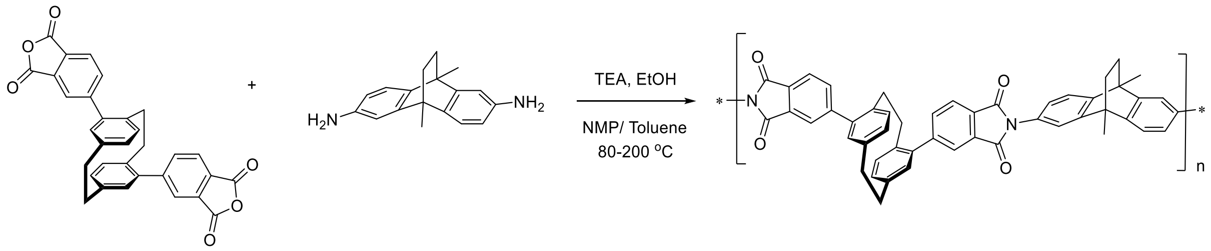

General procedure A was followed using EA(Me) (0.264 g, 0.99 mmol), PCP-BA (0.501 g, 0.99 mmol), ethanol (10 mL), triethylamine (0.708 g, 0.970 mL, 7 mmol), and NMP:toluene (4:1) (5 mL) to afford PCP-PI4 (0.550 g, 76 %) as a pale brown powder.

ν_{max} (cm^{-1}): 2959, 2930, 2859, 1774, 1715, 1361, 1085, 819.

^{1}H NMR (500 MHz, CDCl3): δ 8.14 – 7.96 (br m, 4H, ArH), 7.85 (br s, 2H, ArH), 7.53 –7.42 (br m, 2H, ArH), 7.39 (br s, 2H, ArH), 7.27 (br s, 2H, ArH), 6.82 (br s, 2H, ArH), 6.76 – 6.56 (br m, 4H, ArH), 3.30 (br s, 2H, CH2), 3.16 (br s, 4H, CH2), 2.63 (br s, 2H, CH2), 2.12 – 1.90 (br m, 6H, CH3), 1.74 (br s, 4H, CH2).

BET surface area (CO_2, 195K) = 400 m^2/g; total pore volume 0.3851 cm^3/g at (P/Po = 0.98).

PCP-PI5 from 6-FDA and pseudo-*para*-PCP-dianiline (PCP-*p*-DA)

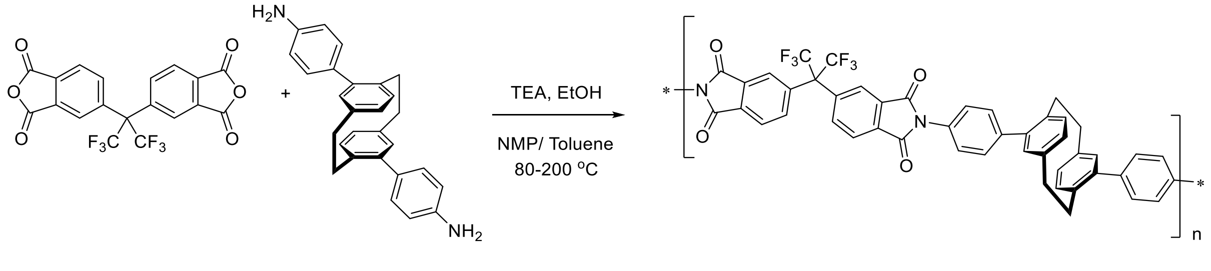

General procedure A was followed using PCP-*p*DA (0.352 g, 0.90 mmol), 4,4-(hexafluoroisopropylidene)-diphthalic anhydride (6-FDA) (0.400 g, 0.90 mmol),), ethanol (10

mL), triethylamine (0.455 g, 0.630 mL, 4.5 mmol), and NMP:toluene (4:1) (5 mL) to afford PCP-PI5 (0.370 g, 51 %) as a pale brown powder.

ν_{max} (cm^{-1}): 2926, 1784, 1715, 1369, 1208, 1192, 836, 721.

BET surface area (CO_2, 195K) = 285 m^2/g; total pore volume 0.2783 cm^3/g at (P/Po = 0.98).

3 Qualitative Analysis

3.1 MALDI-TOF analysis

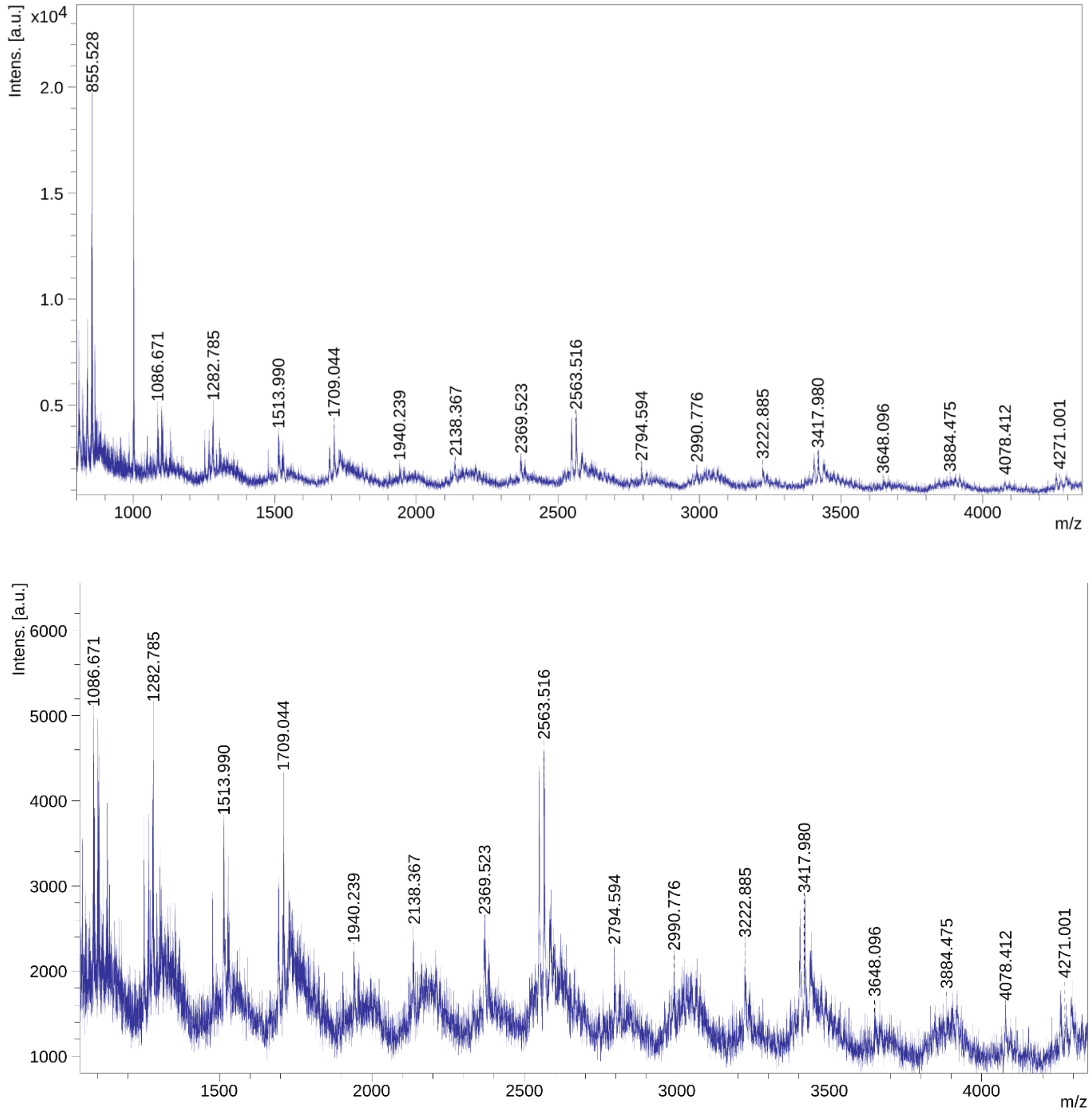

Figure S2. MALDI-TOF spectrum of repeating units of **PCP-PI2**, shown at different y-scales for clarity.

3.2 Solid-state NMR spectra of all PCP-PIs

3.2.1 **Table S1**. Solid-state NMR experimental parameters.

Sample	Nucleus	Figure	Pulse sequence	T_1 (s)	Recycle delay (s)	Number of scans	Experiment time
PCP-PI1	^{1}H	3d	echo	0.2	2	8	0.3 min
	^{13}C	3a	CP	-	0.3	1688	8 min
	^{15}N	3b	CP	-	0.3	246328	20.5 h
PCP-PI2	^{1}H	3d	echo	0.3	2	8	0.3 min
	^{13}C	3a	CP	-	0.4	2624	17 min
	^{15}N	3b	CP	-	0.3	883680	73.6 h
PCP-PI3	^{1}H	3d	echo	0.3	2	8	0.3 min
	^{13}C	3a	CP	-	0.4	8904	59 min
	^{15}N	3b	CP	-	0.3	491272	40.9 h
	^{19}F	3c	echo	0.9	2	32	1 min
PCP-PI4	^{1}H	3d	echo	0.2	2	8	0.3 min
	^{13}C	3a	CP	-	0.3	18420	92 min
	^{15}N	3b	CP	-	0.25	308852	21.4 h
PCP-PI5	^{1}H	3d	echo	0.2	2	8	0.3 min
	^{13}C	3a	CP	-	0.3	9676	48 min
	^{15}N	3b	CP	-	0.25	233980	16.2 h
	^{19}F	3c	echo	0.6	2	32	1 min

3.2.2 NMR spectra of **PCP-PI1**

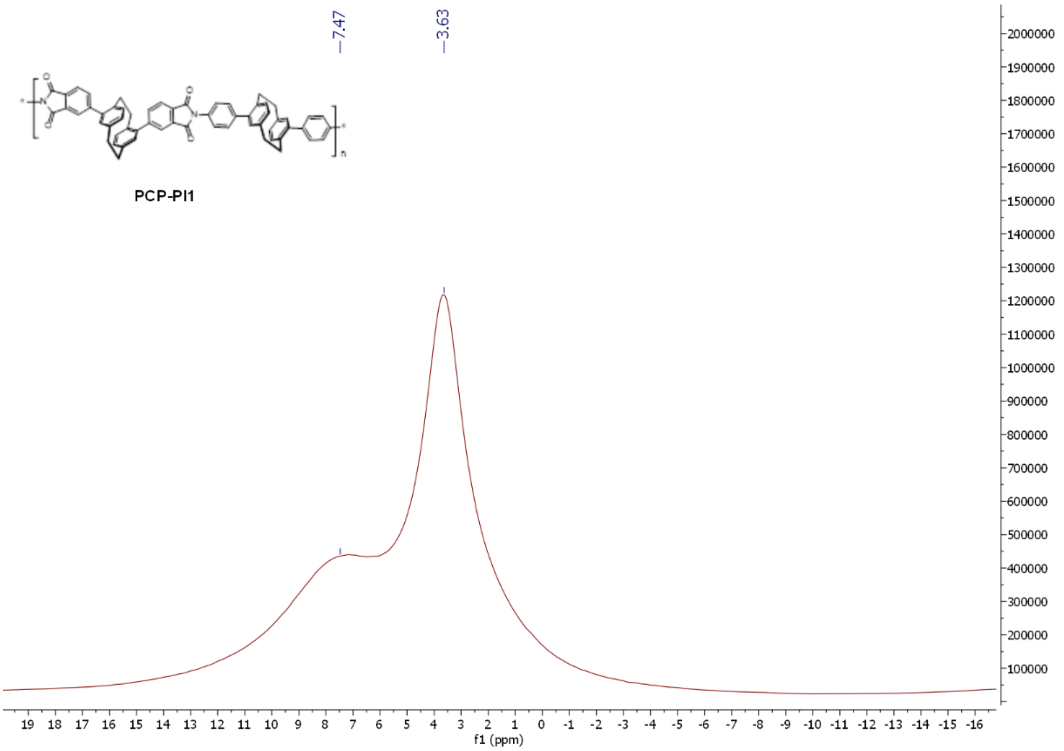

Figure S3. ^{1}H solid-state MAS NMR spectrum of **PCP-PI1**.

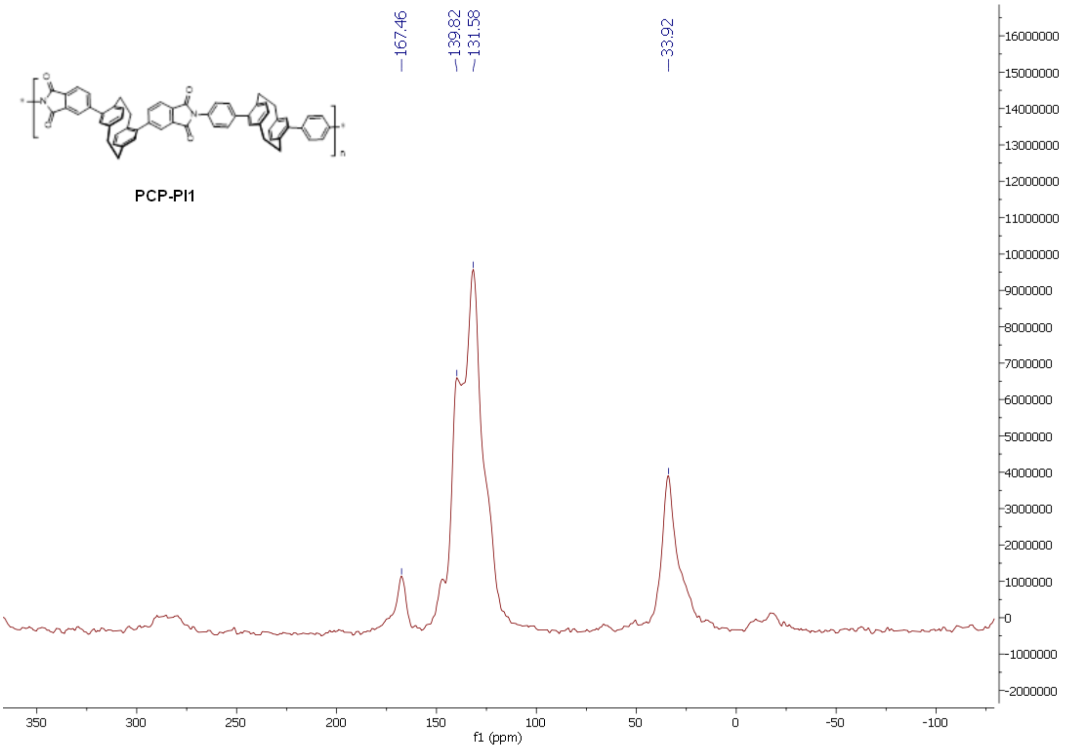

Figure S4. ^{1}H-^{13}C CP solid-state MAS NMR spectrum of **PCP-PI1**.

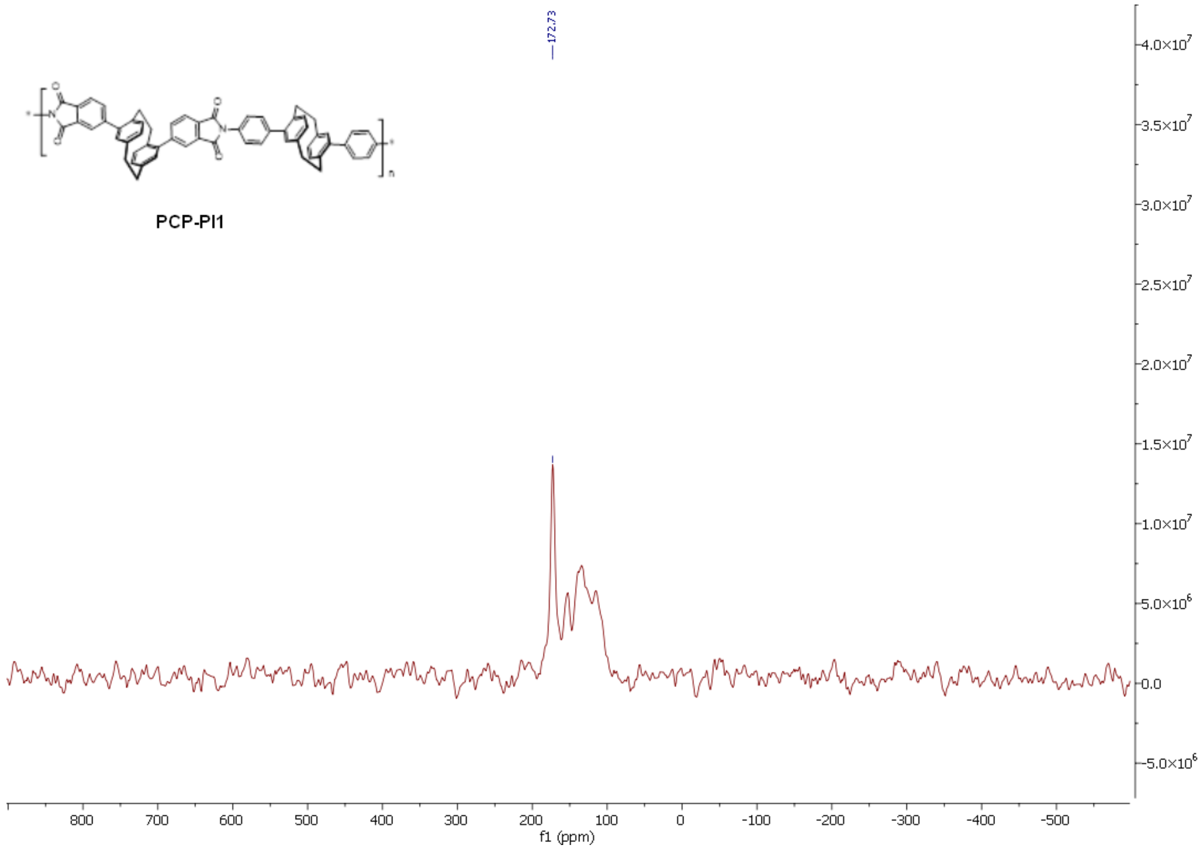

Figure S5. ^{1}H-^{15}N CP solid-state MAS NMR spectrum of **PCP-PI1**.

3.2.3 NMR spectra of **PCP-PI2**

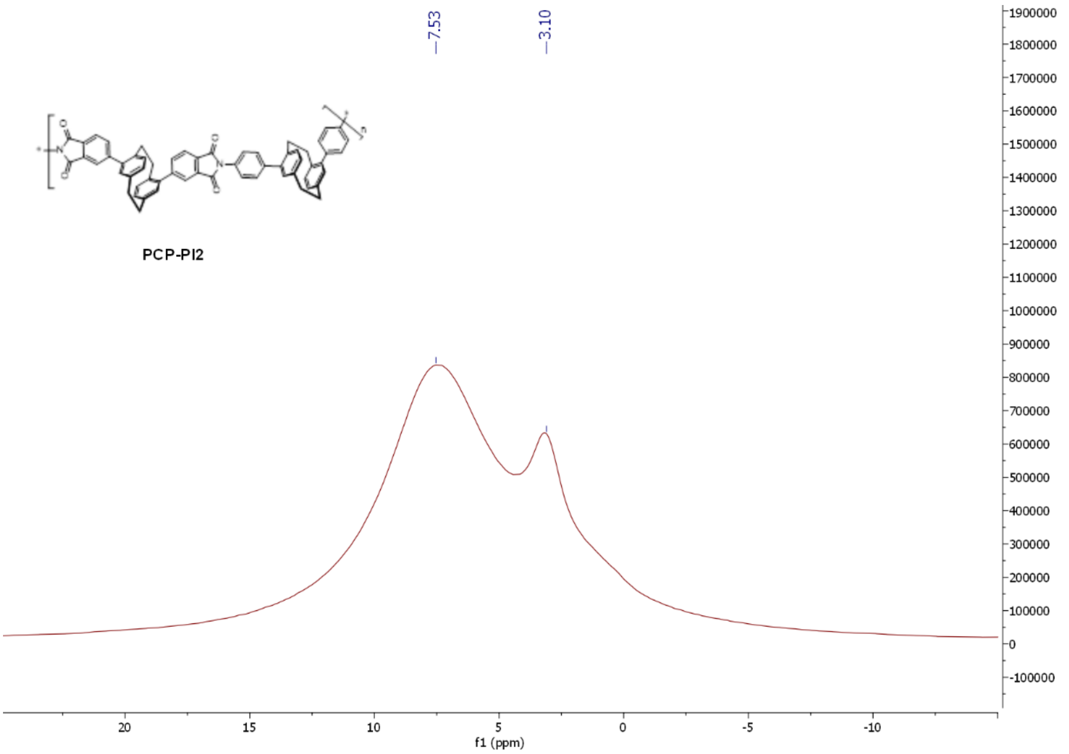

Figure S6. ^{1}H solid-state MAS NMR spectrum of **PCP-PI2**.

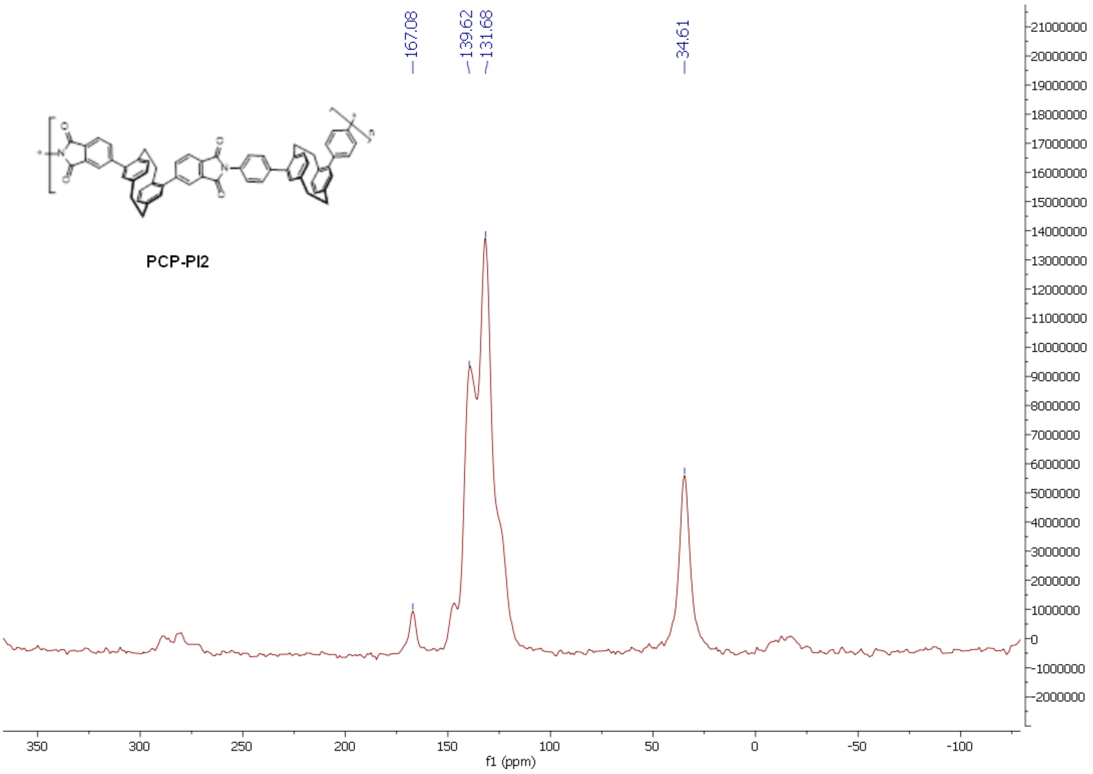

Figure S7. ^{1}H-^{13}C CP solid-state MAS NMR spectrum of **PCP-PI2**.

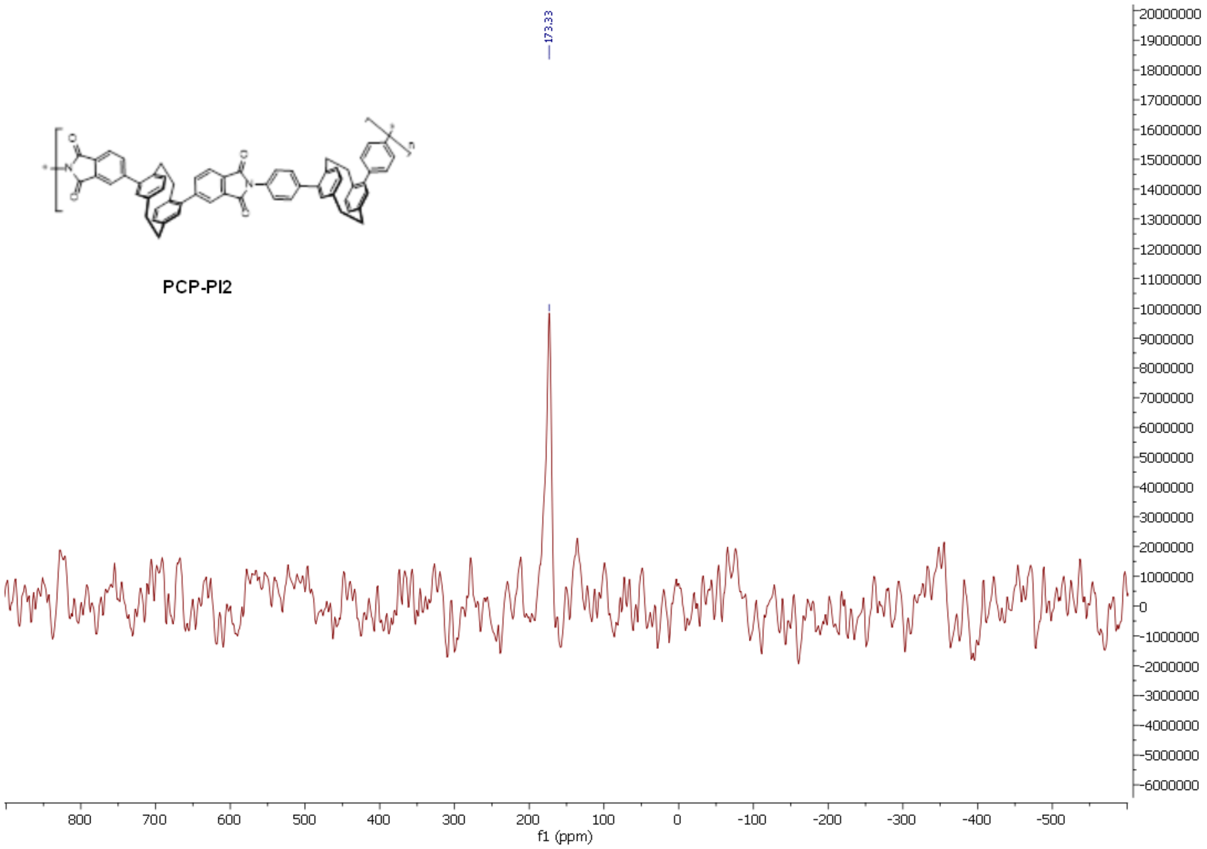

Figure S8. ^{1}H-^{15}N CP solid-state MAS NMR spectrum of **PCP-PI2**.

3.2.4 NMR spectra of **PCP-PI3**

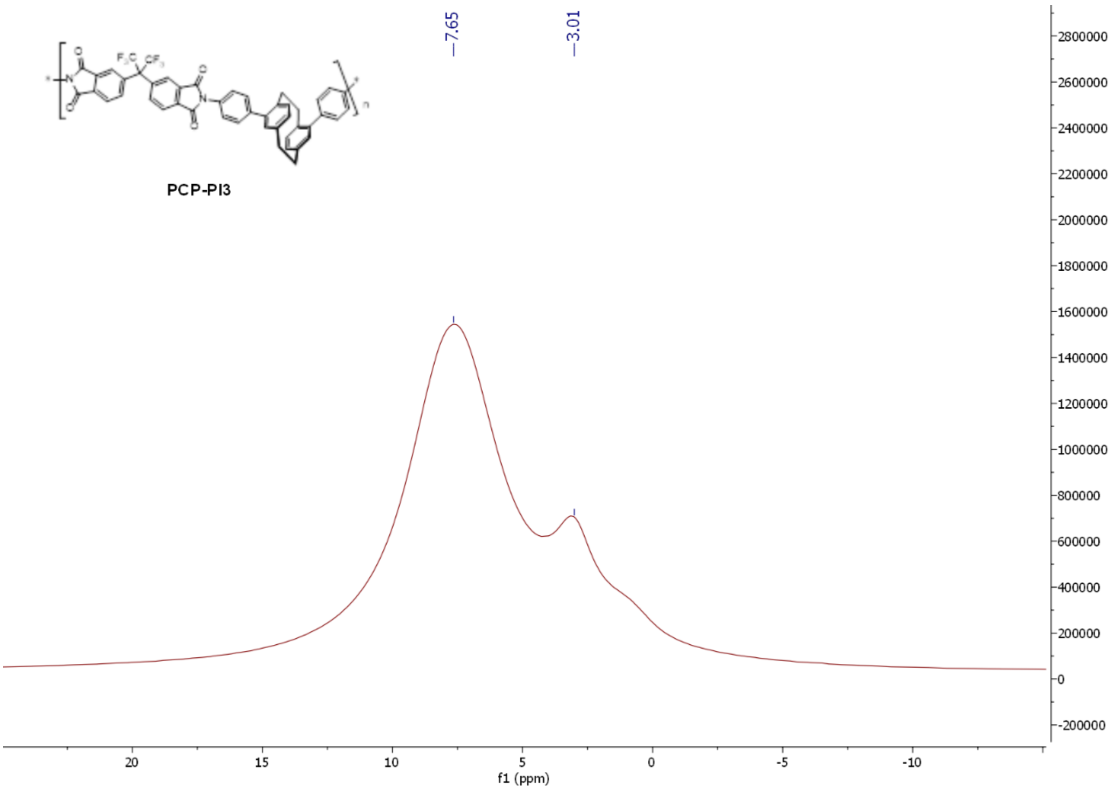

Figure S9. ^{1}H solid-state MAS NMR spectrum of **PCP-PI3**.

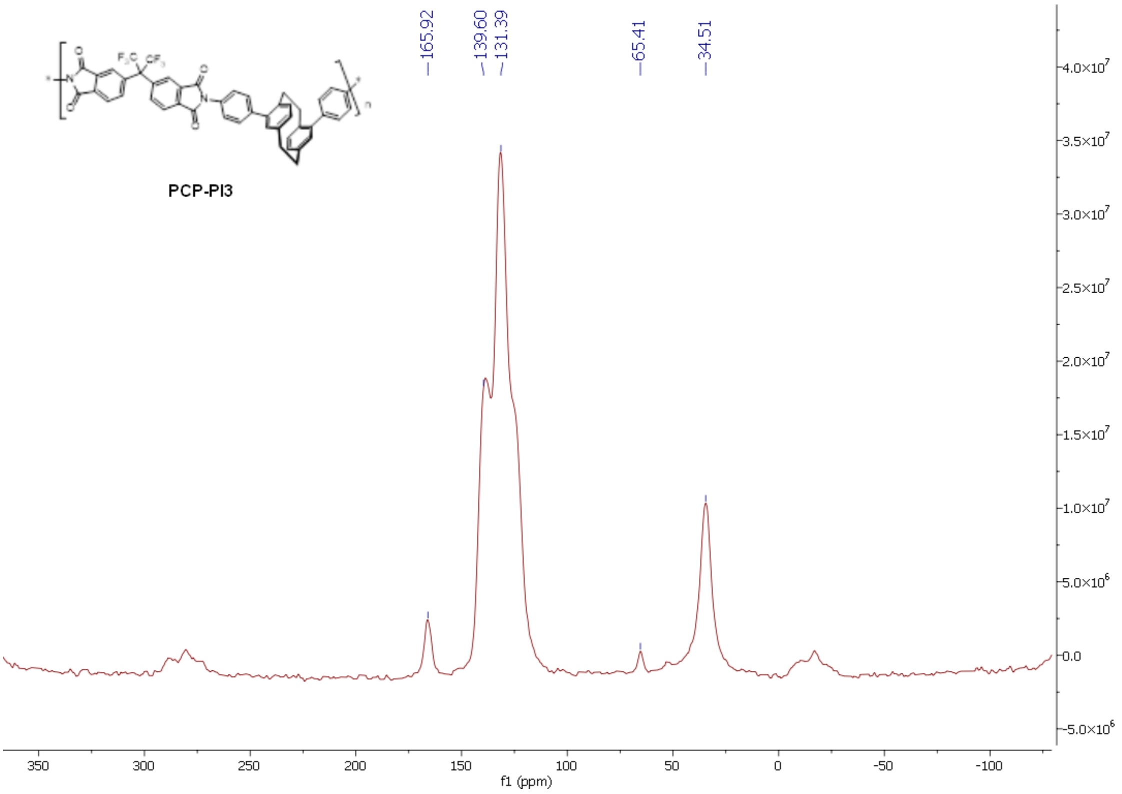

Figure S10. ^{1}H-^{13}C CP solid-state MAS NMR spectrum of **PCP-PI3**.

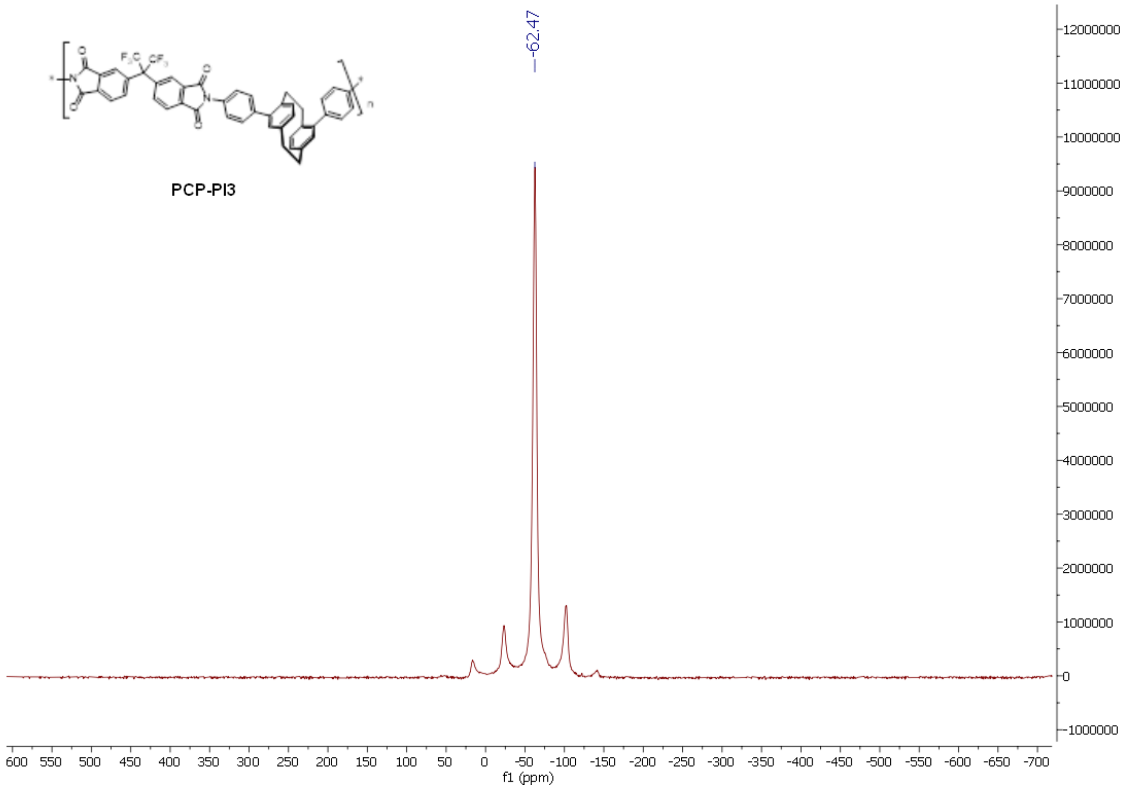

Figure S11. ^{19}F solid-state MAS NMR spectrum of **PCP-PI3**.

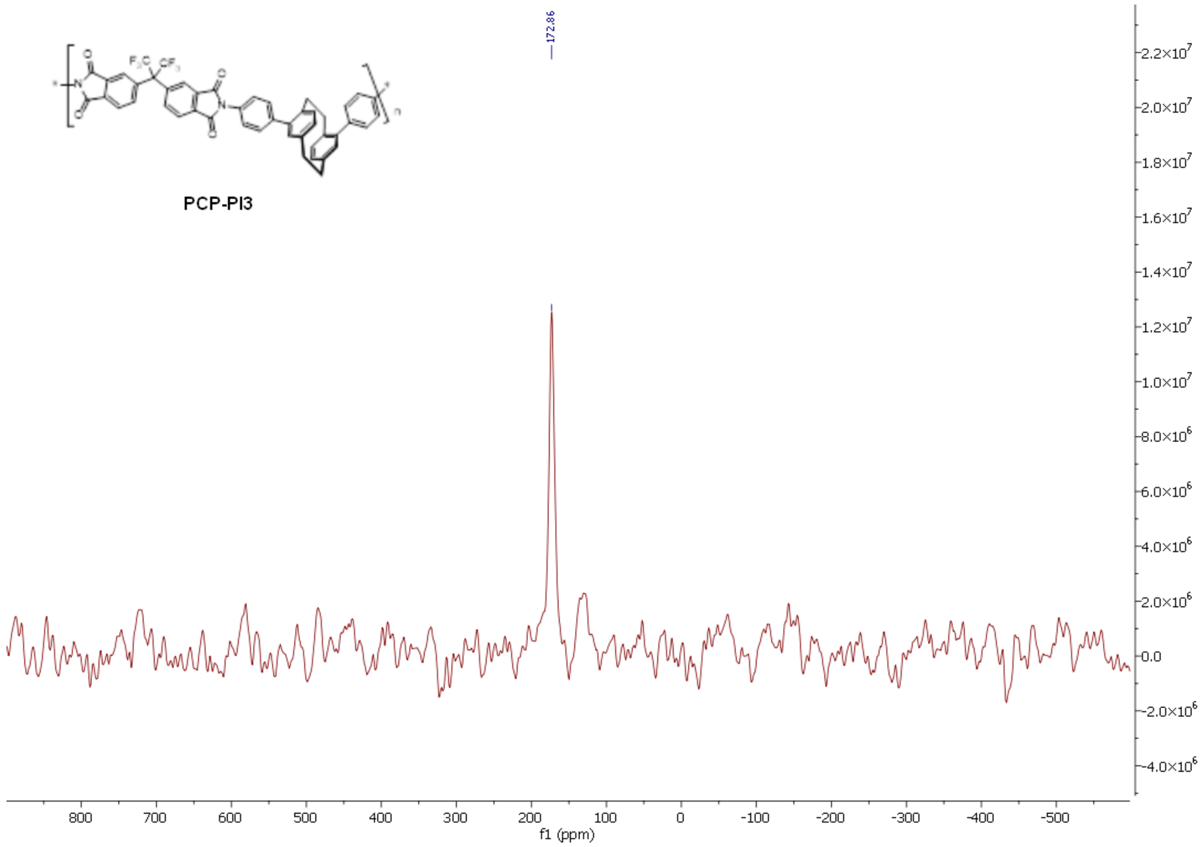

Figure S12. ^{1}H-^{15}N CP solid-state MAS NMR spectrum of **PCP-PI3**.

3.2.5 NMR spectra of **PCP-PI4**

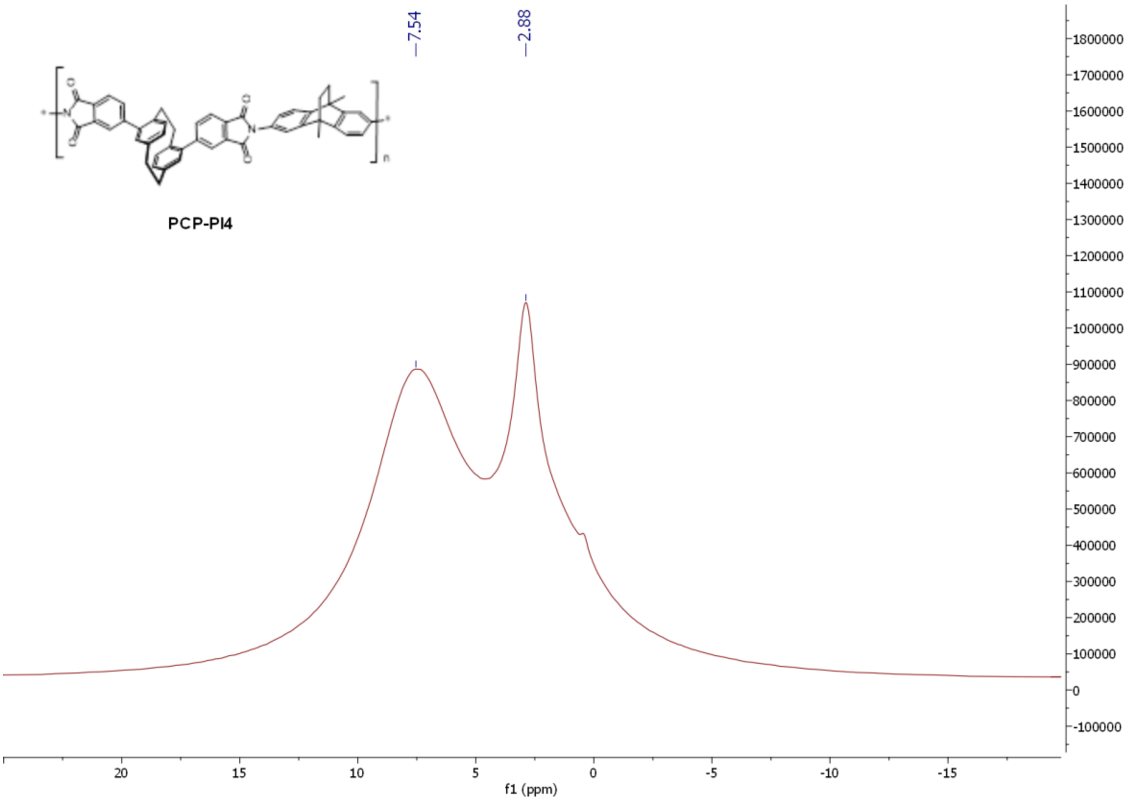

Figure S13. ^{1}H solid-state MAS NMR spectrum of **PCP-PI4**.

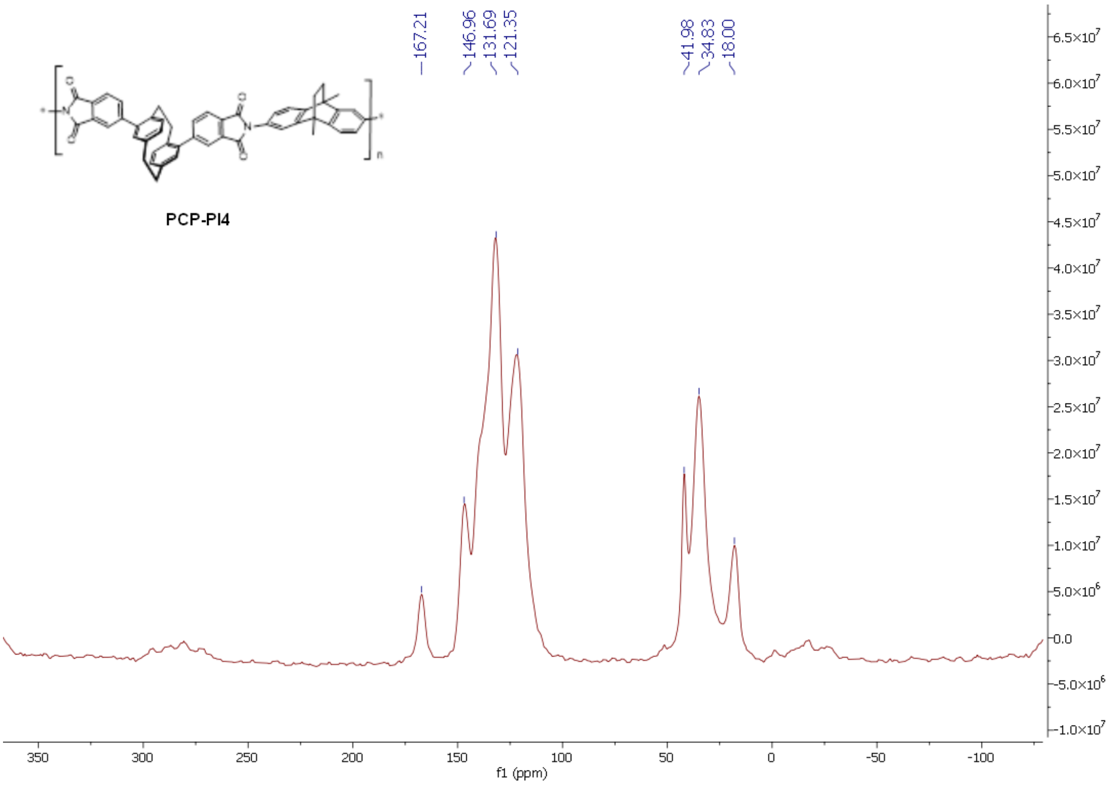

Figure S14. ^{1}H-^{13}C CP solid-state MAS NMR spectrum of **PCP-PI4**.

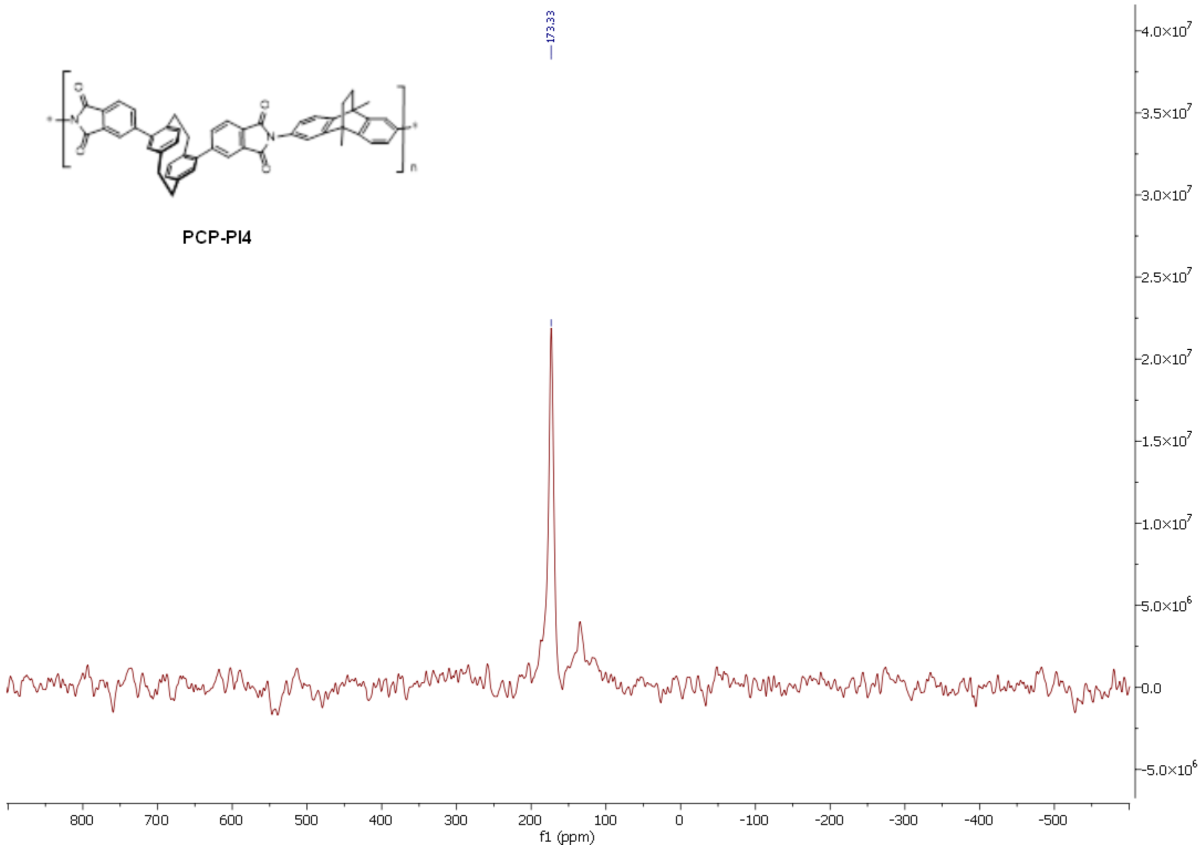

Figure S15. ^{1}H-^{15}N CP solid-state MAS NMR spectrum of **PCP-PI4**.

3.2.6 NMR spectra of **PCP-PI5**

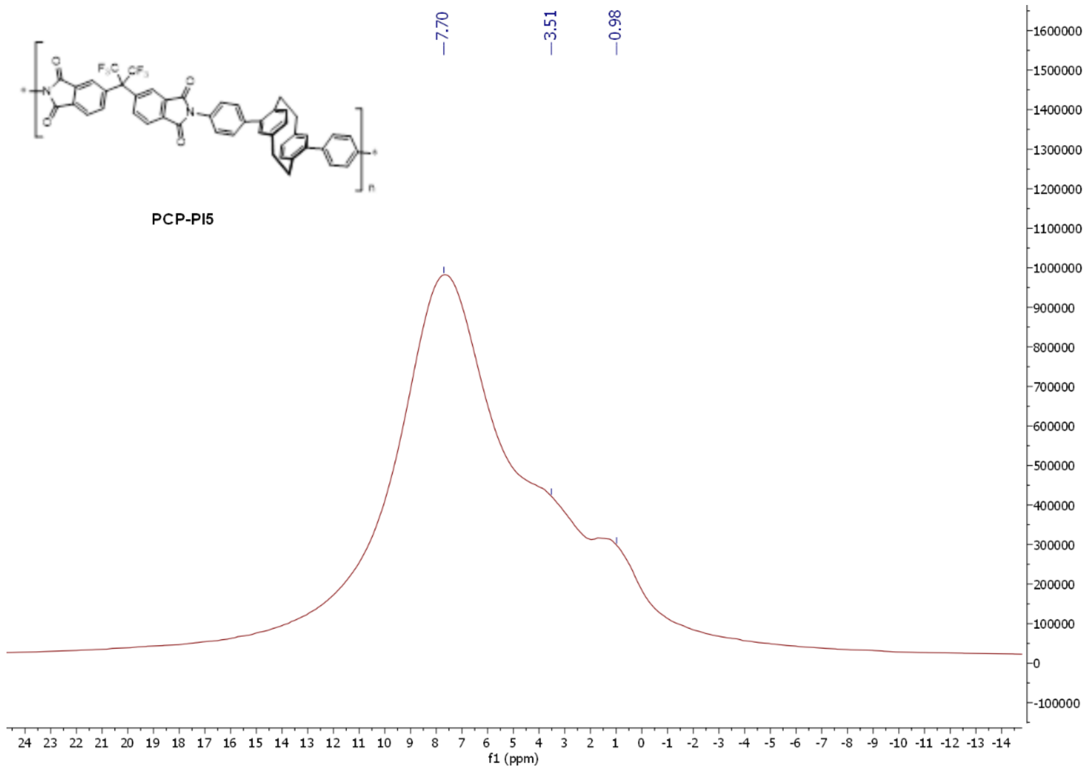

Figure S16. ^{1}H solid-state MAS NMR spectrum of **PCP-PI5**.

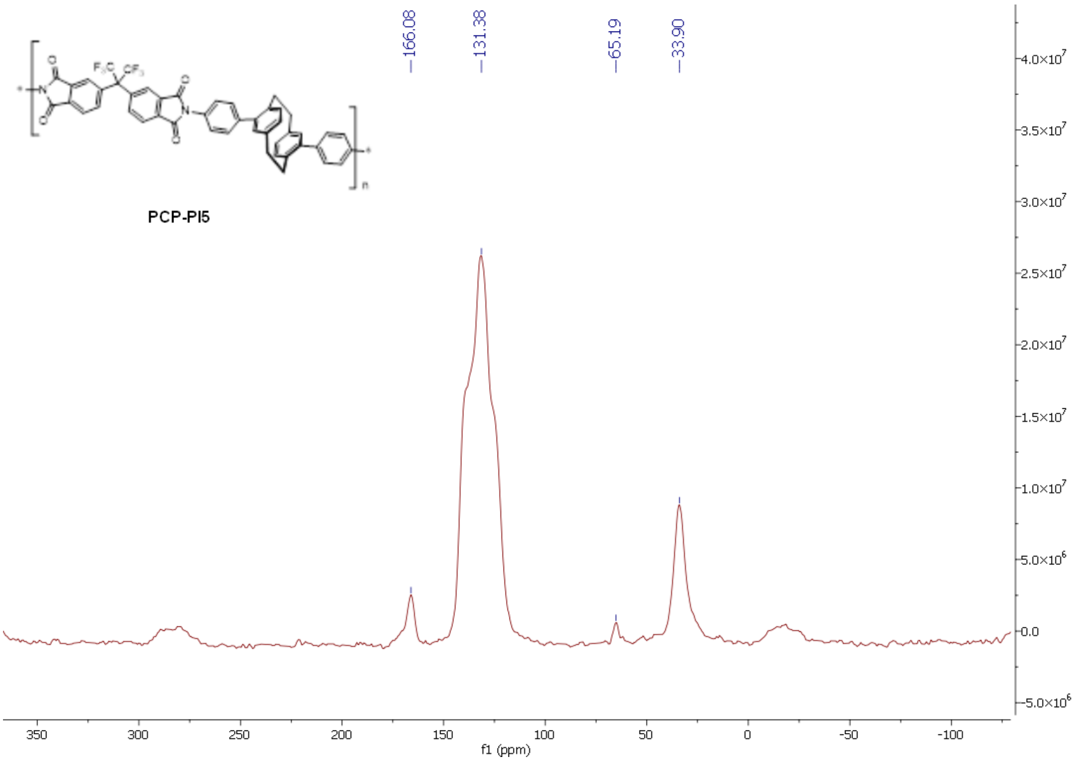

Figure S17. ^{1}H-^{13}C CP solid-state MAS NMR spectrum of **PCP-PI5**.

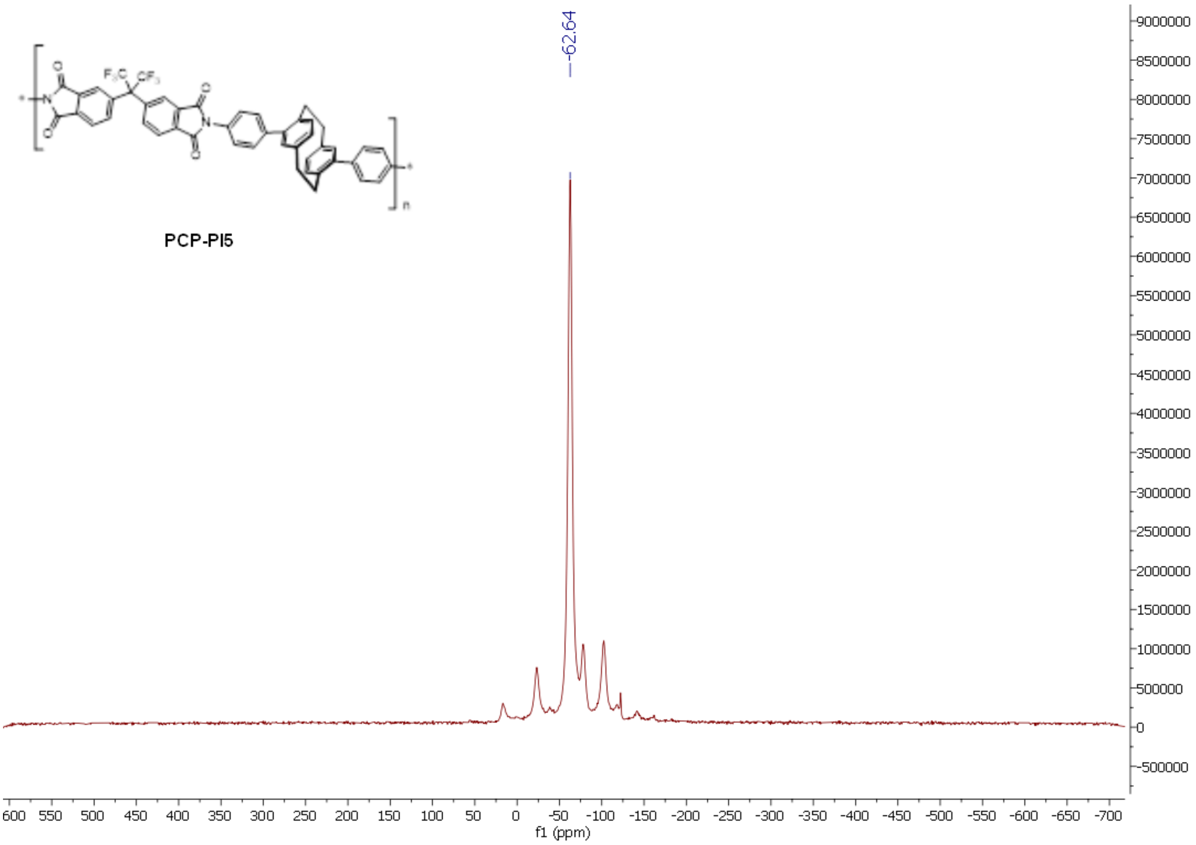

Figure S18. ^{19}F solid-state MAS NMR spectrum of **PCP-PI5**.

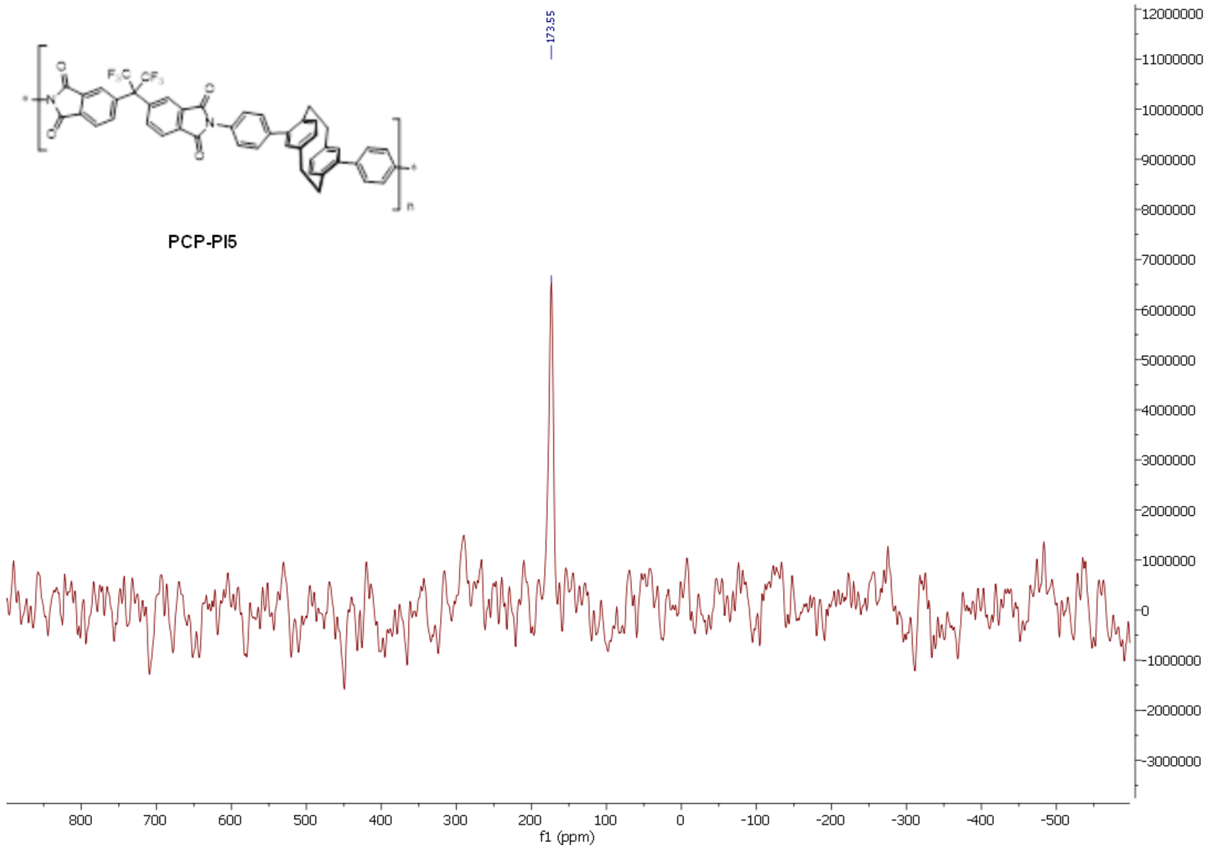

Figure S19. ^{1}H-^{15}N CP solid-state MAS NMR spectrum of **PCP-PI5**.

3.3 FT-IR Spectra of Polymers

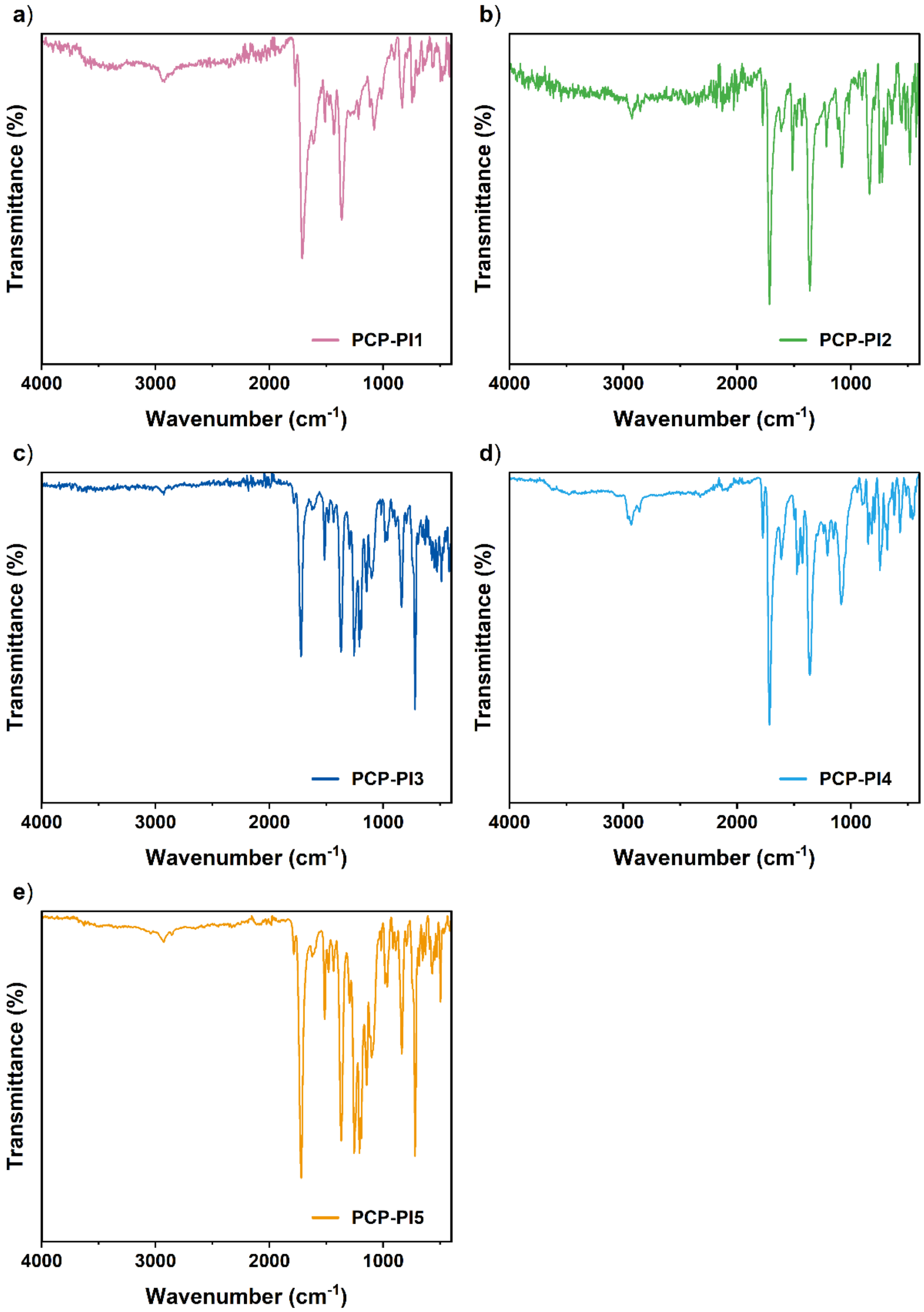

Figure S20. FT-IR spectra of the PIs: (a) **PCP-PI1**, (b) **PCP-PI2**, (c) **PCP-PI3**, (d) **PCP-PI4**. and (e) **PCP-PI5**.

3.4 TG and DTG analysis

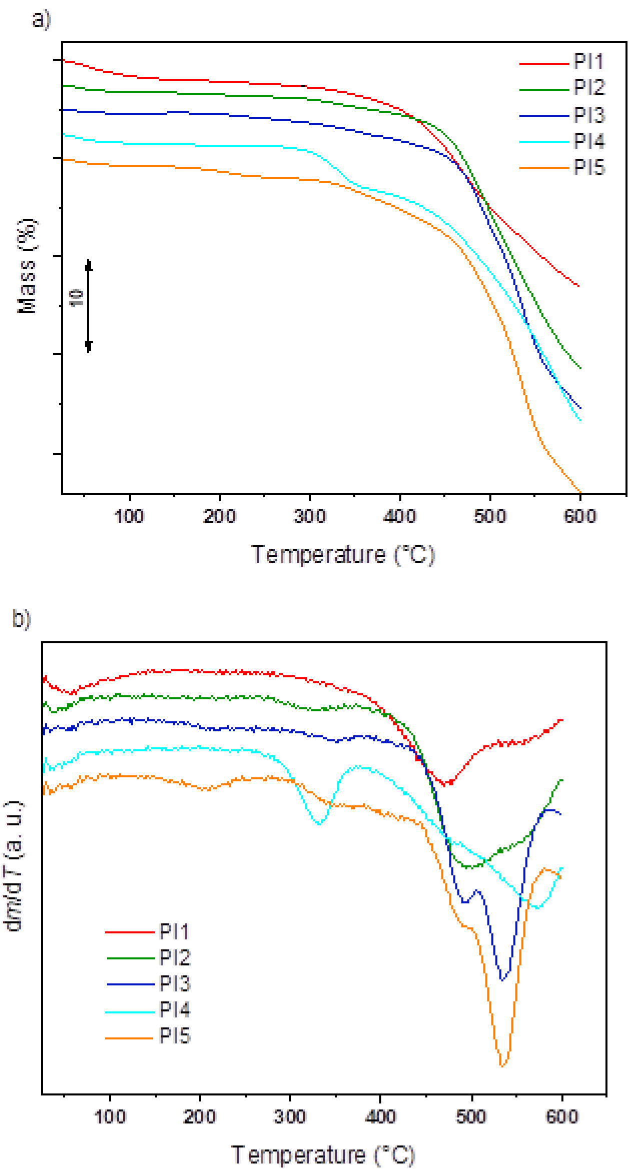

Figure S21. (a) TG and (b) DTG curves of the PIs performed under nitrogen atmosphere.

Table S2. Mass losses, prior main decomposition (temperature ranges were determined based on DTG curves), and onset decomposition temperatures of the polymers.

Polymer	Temperature range; Δm	Onset decomposition temperate (°C)
**PCP-PI1**		385
**PCP-PI2**	285-370 °C; 1,22%	415
**PCP-PI3**	330-370 °C; 1,49%	440
**PCP-PI4**	285-370 °C; 4,24%	410
**PCP-PI5**	160-255 °C; 1,08%<br>300-435 °C; 4,66%	440

4 Porosity and Selectivity Measurements of PCP-PIs

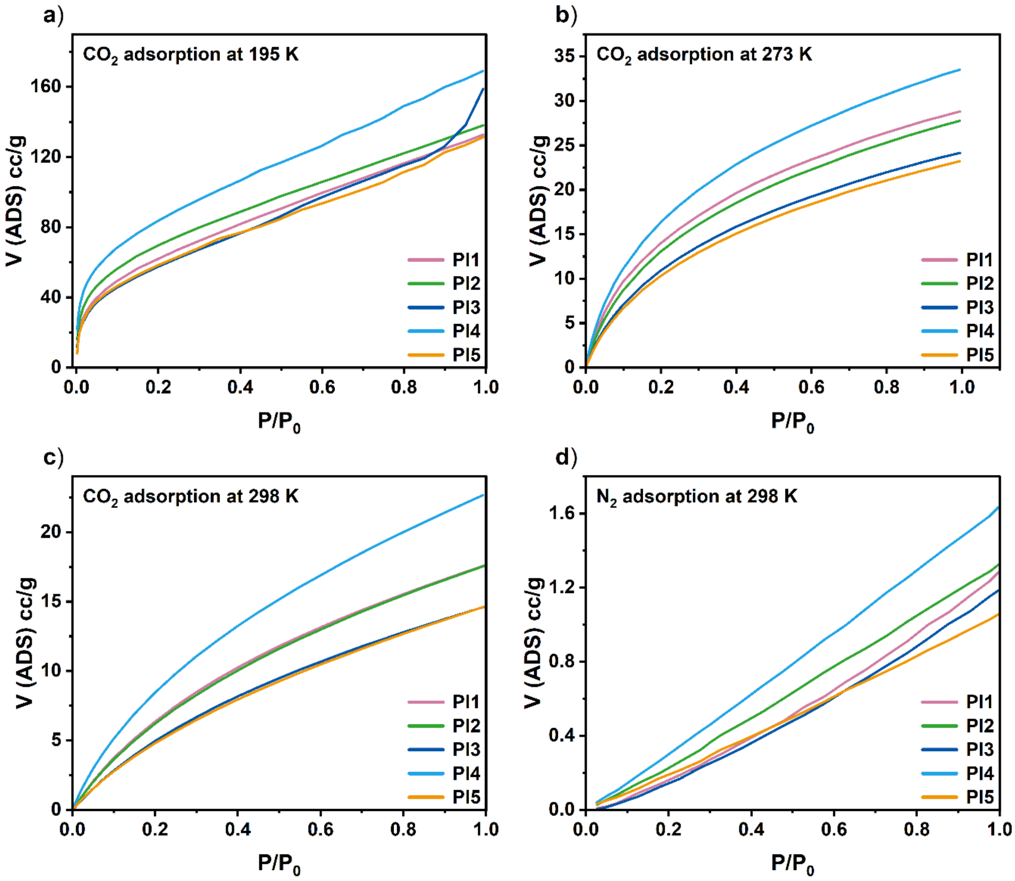

Figure S22. Gas adsorption isotherms of PCP-based polyimides (**PCP-PI1-PI5**): (a) CO_2 adsorption at 195 K; (b) CO_2 adsorption at 273 K; (c) CO_2 adsorption at 298 K; (d) N_2 adsorption at 298 K.

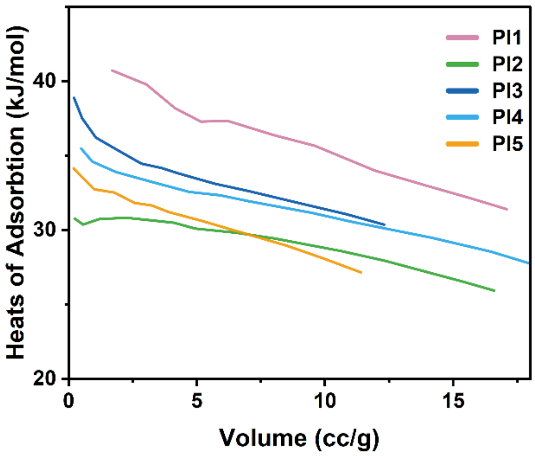

Figure S23. Isosteric heat of adsorption (in kJ mol^{-1}) of corresponding gas at zero coverage in polymers **PCP-PI1-PI5** calculated from isotherms collected at 273 and 298 K and fitted with the Langmuir-Freundlich equation and calculated *via* the Clausius Clapeyron equation.

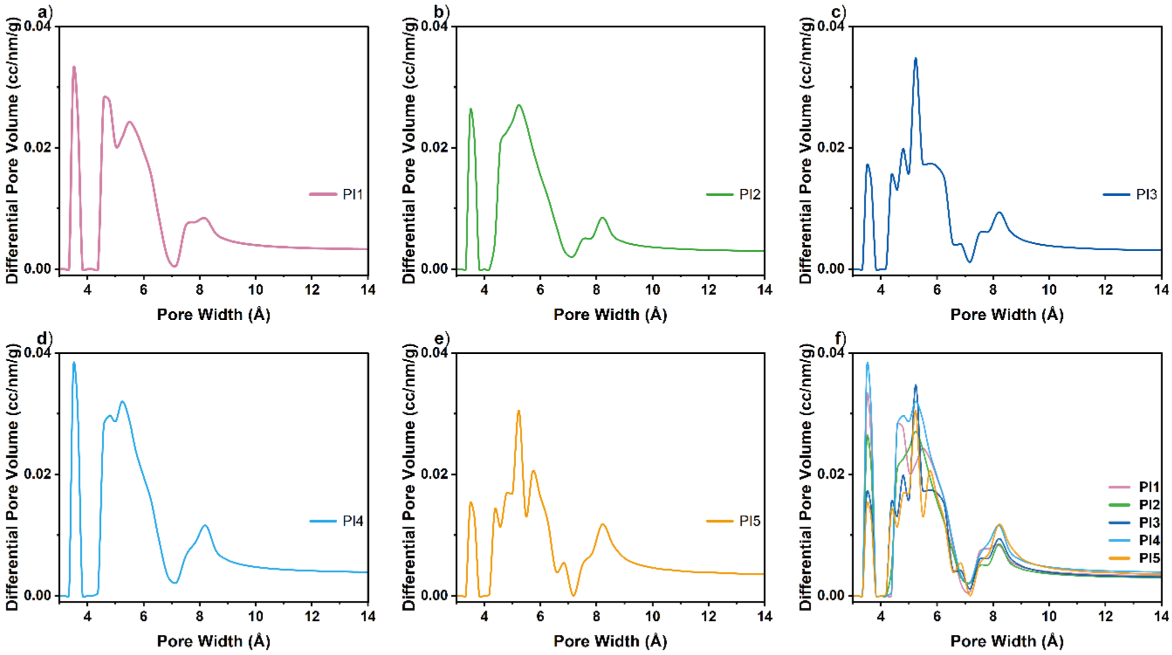

Figure S24. Pore-size distribution (PSD) curves of PCP-based polyimides (PCP-**PI1-PI5**) derived from CO_2 adsorption at 273 K: (a) **PCP**-PI1; (b) **PCP**-PI2; (c) **PCP**-PI3; (d) **PCP**-PI4; (e) **PCP**-PI5; (f) Comparison of all samples.

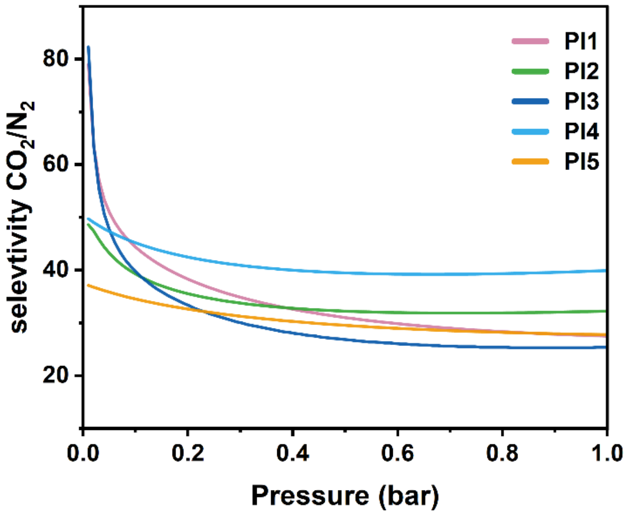

Figure S25. Selectivity of CO_2 over N_2 adsorption (IAST, 298 K for a CO_2/N_2 15/85 composition).

5 Wide-angle X-ray diffraction (WAXD) analyses

Wide-angle X-ray diffraction (WAXD) analyses of the PCP-PI1-5 revealed poor crystallinity of the polymers with all exhibiting a mayor peak at around 2θ = 14° and a shoulder at 26°. Based on the interpretation of Zhang et al. that reported similar polymers, these peaks correspond to dominant spacing of interchain distances of 6.3 Å and π-π stacking of the aromatic segments of around 3.4 Å, respectively.[3]

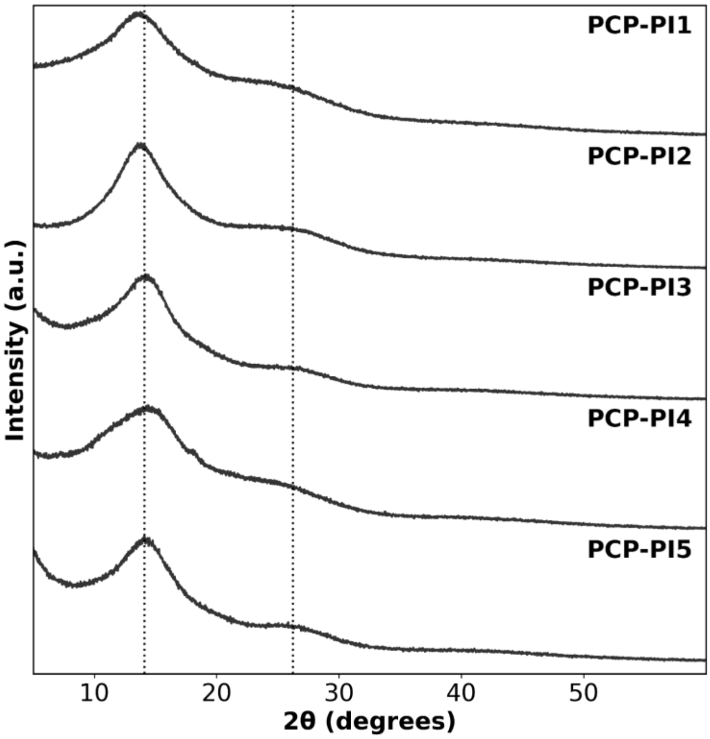

Figure. S26. WAXD diffraction patterns for polymers **PCP-PI1-5** between $2\theta = 5$–60°.

6 SEM Images and EDX mapping of the Polymers

6.1 SEM images

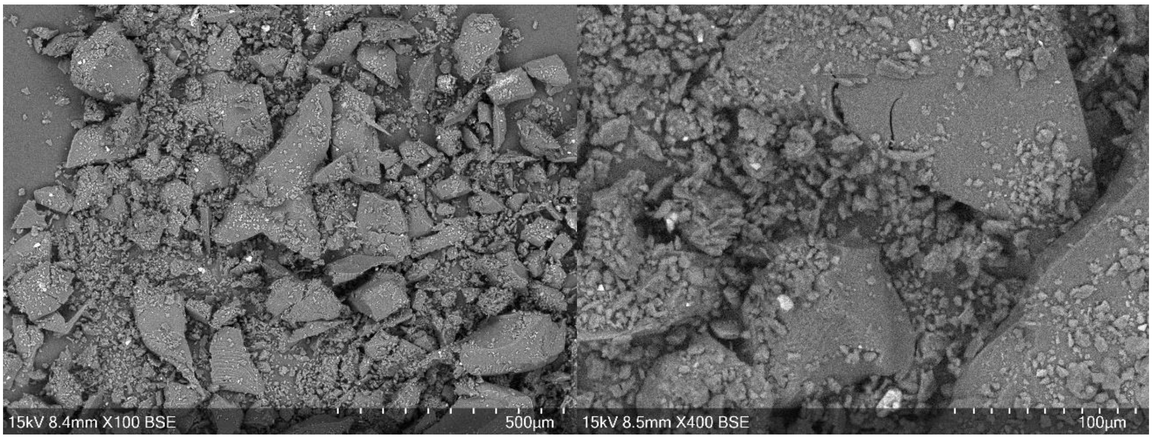

Figure S27. SEM image of a sample of **PCP-PI1** at different magnifications.

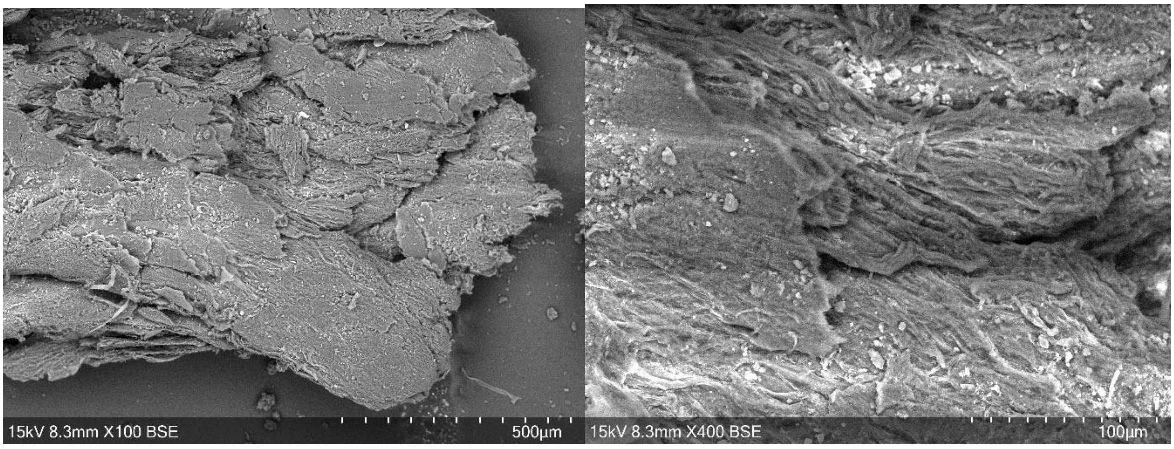

Figure S28. SEM image of a sample of **PCP-PI2** at different magnifications.

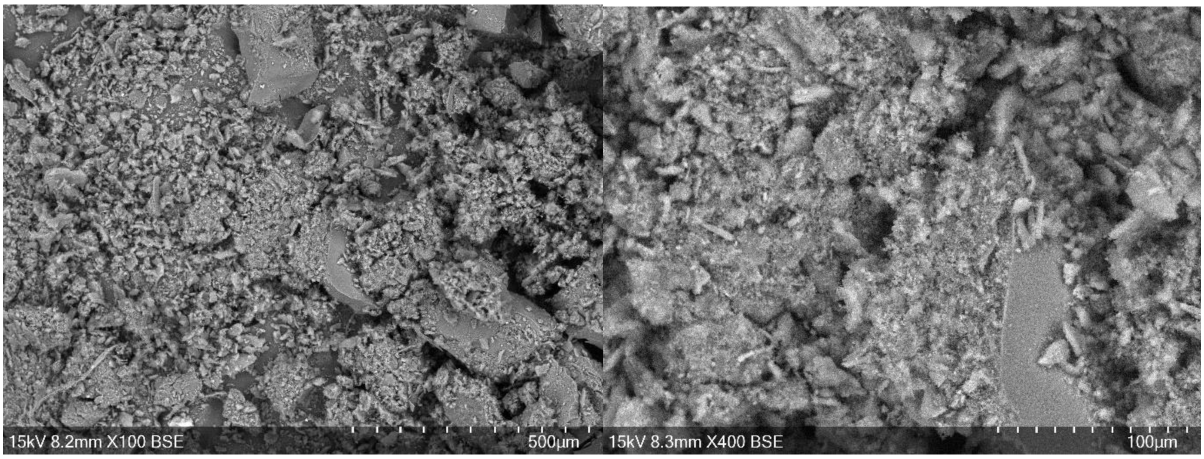

Figure S29. SEM image of a sample of **PCP-PI3** at different magnifications.

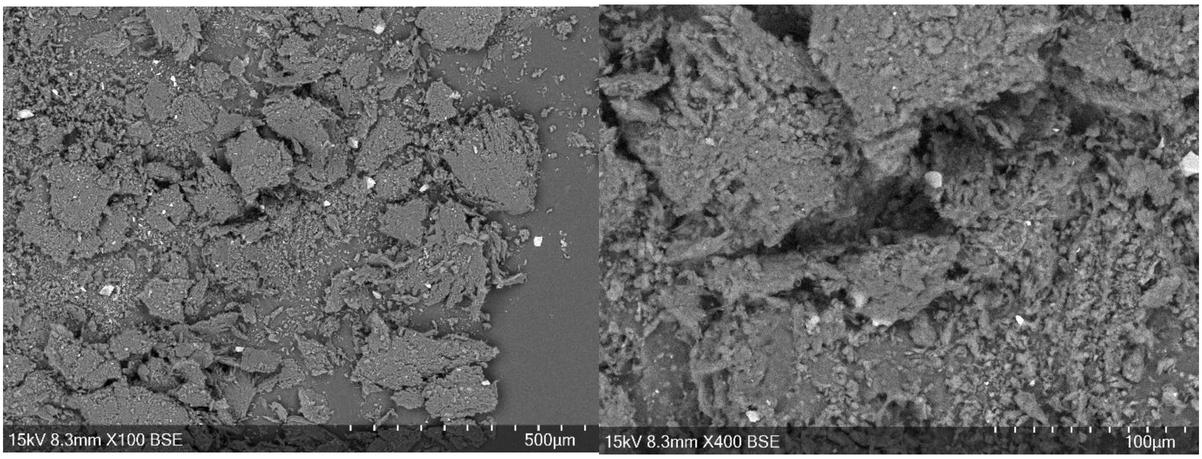

Figure S30. SEM image of a sample of **PCP-PI4** at different magnifications.

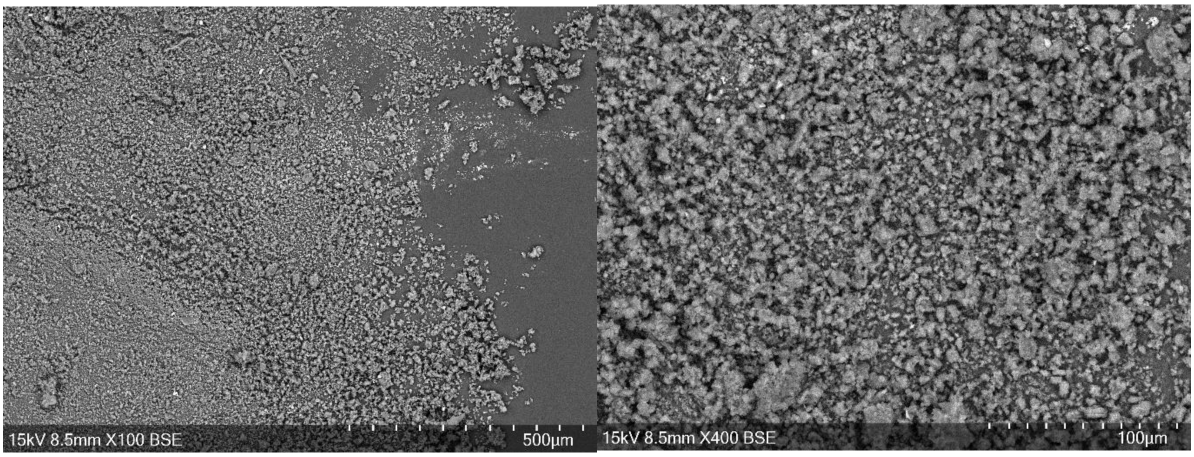

Figure S31. SEM image of a sample of a sample of **PCP-PI5** at different magnifications.

6.2 EDX mapping

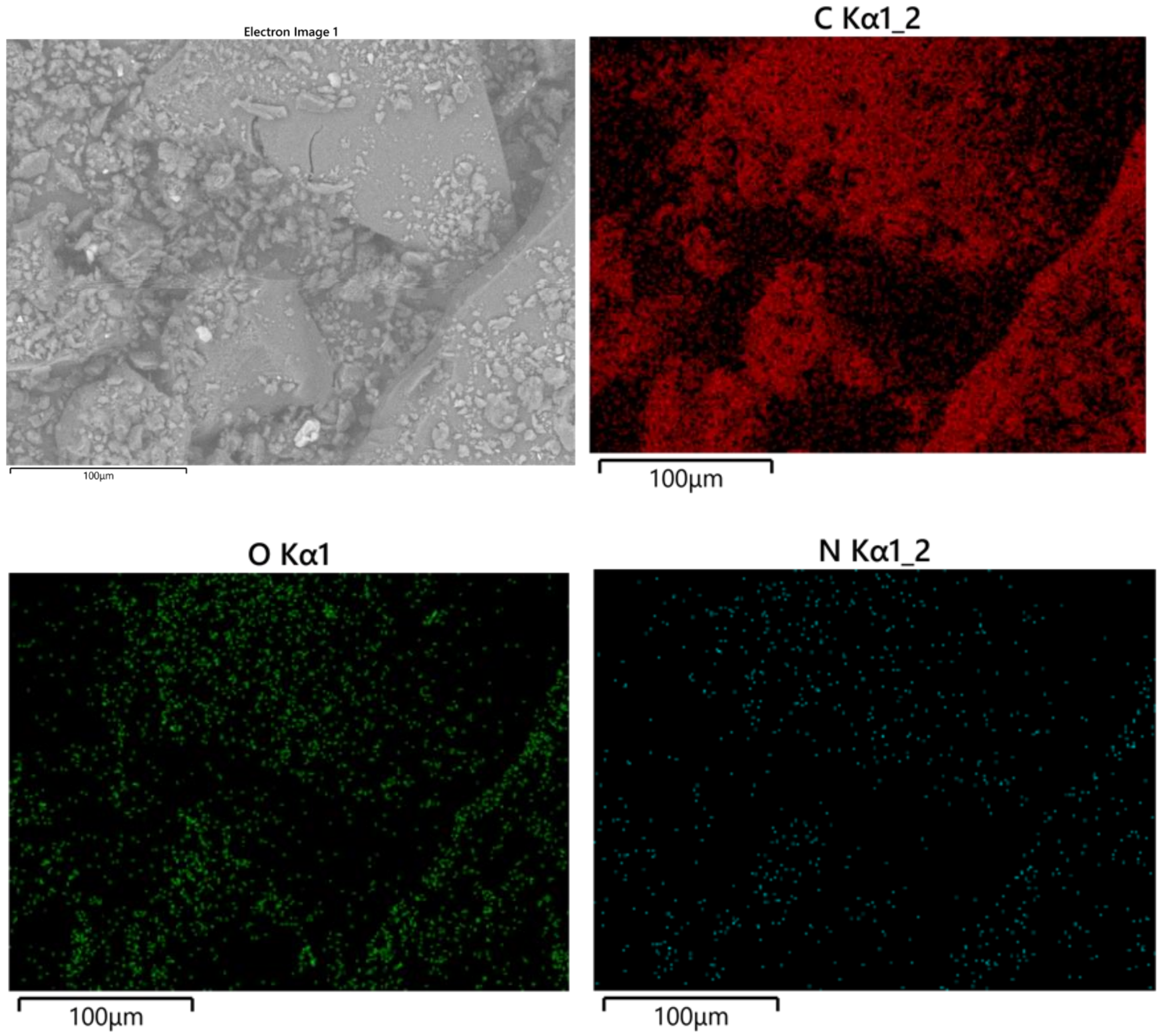

Figure S32. EDX mapping of the elements C (*red*), O (*green*) and N (*blue*) for a sample of **PCP-PI1**.

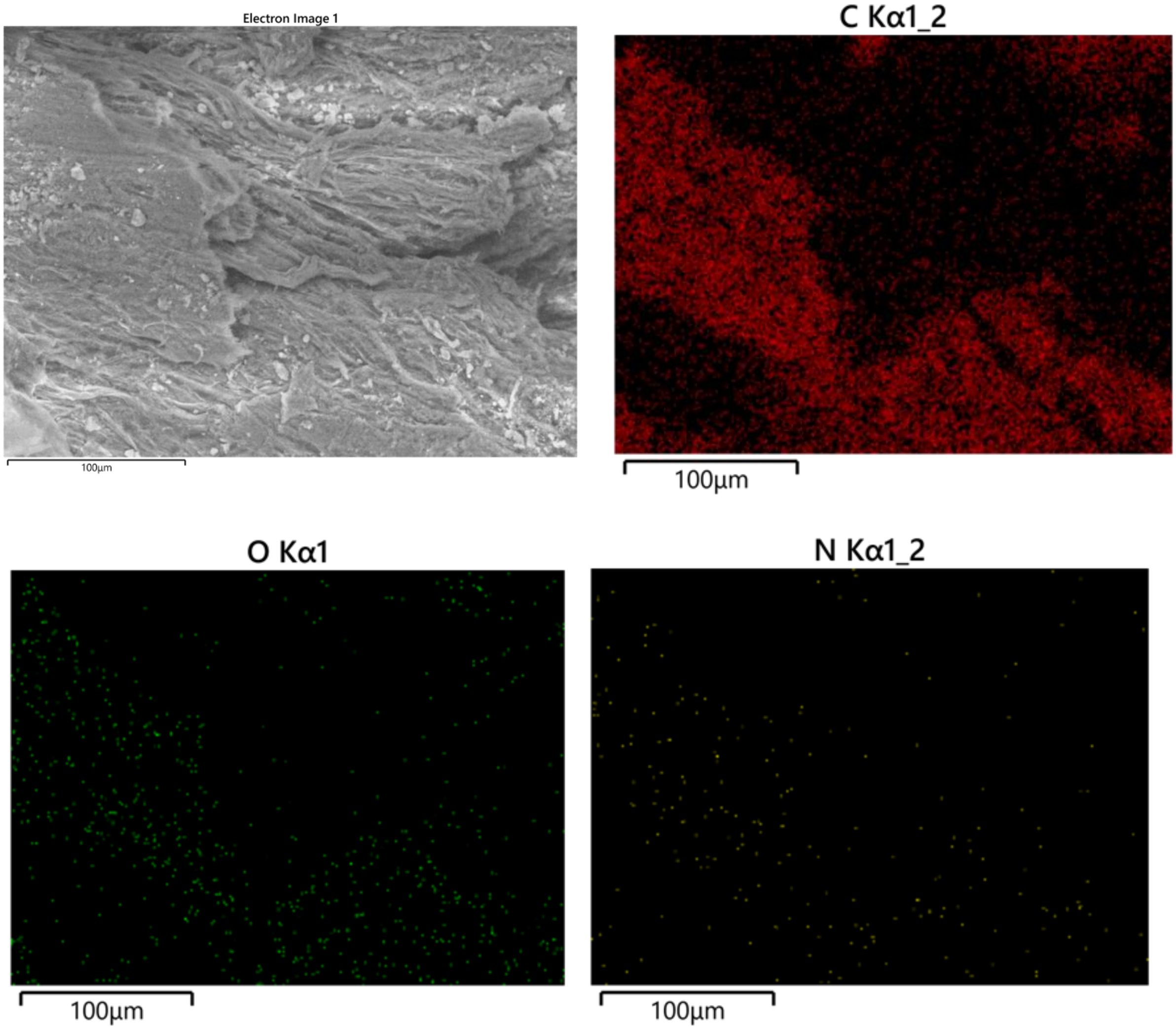

Figure S33. EDX mapping of the elements C (*red*), O (*green*) and N (*yellow*) for a sample of **PCP-PI2**

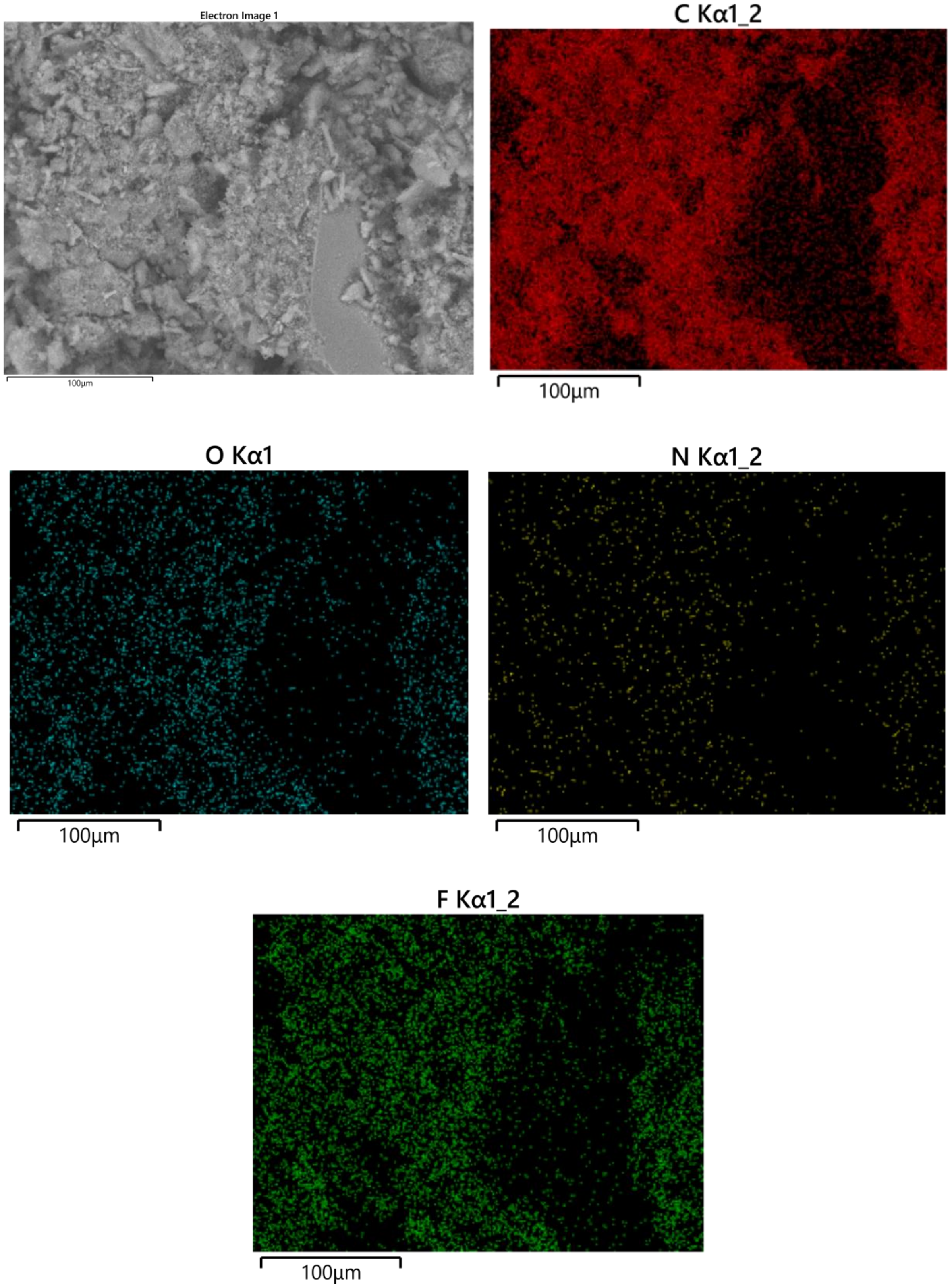

Figure S34. EDX mapping of the elements C (*red*), O (*blue*), N (*yellow*) and F (*green*) for a sample of **PCP-PI3**.

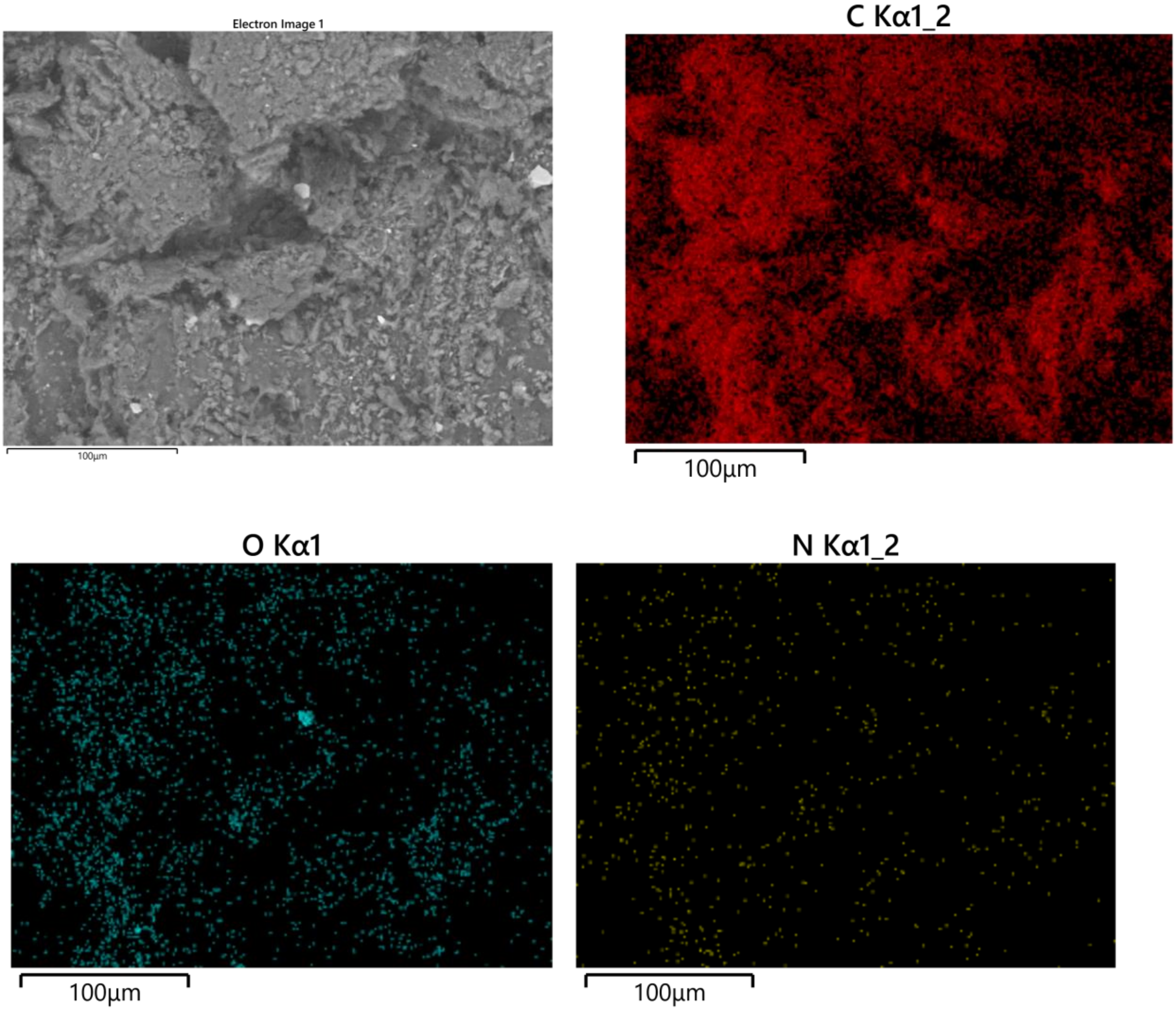

Figure S35. EDX mapping of the elements C (*red*), O (*blue*) and N (*yellow*) for a sample of **PCP-PI4**.

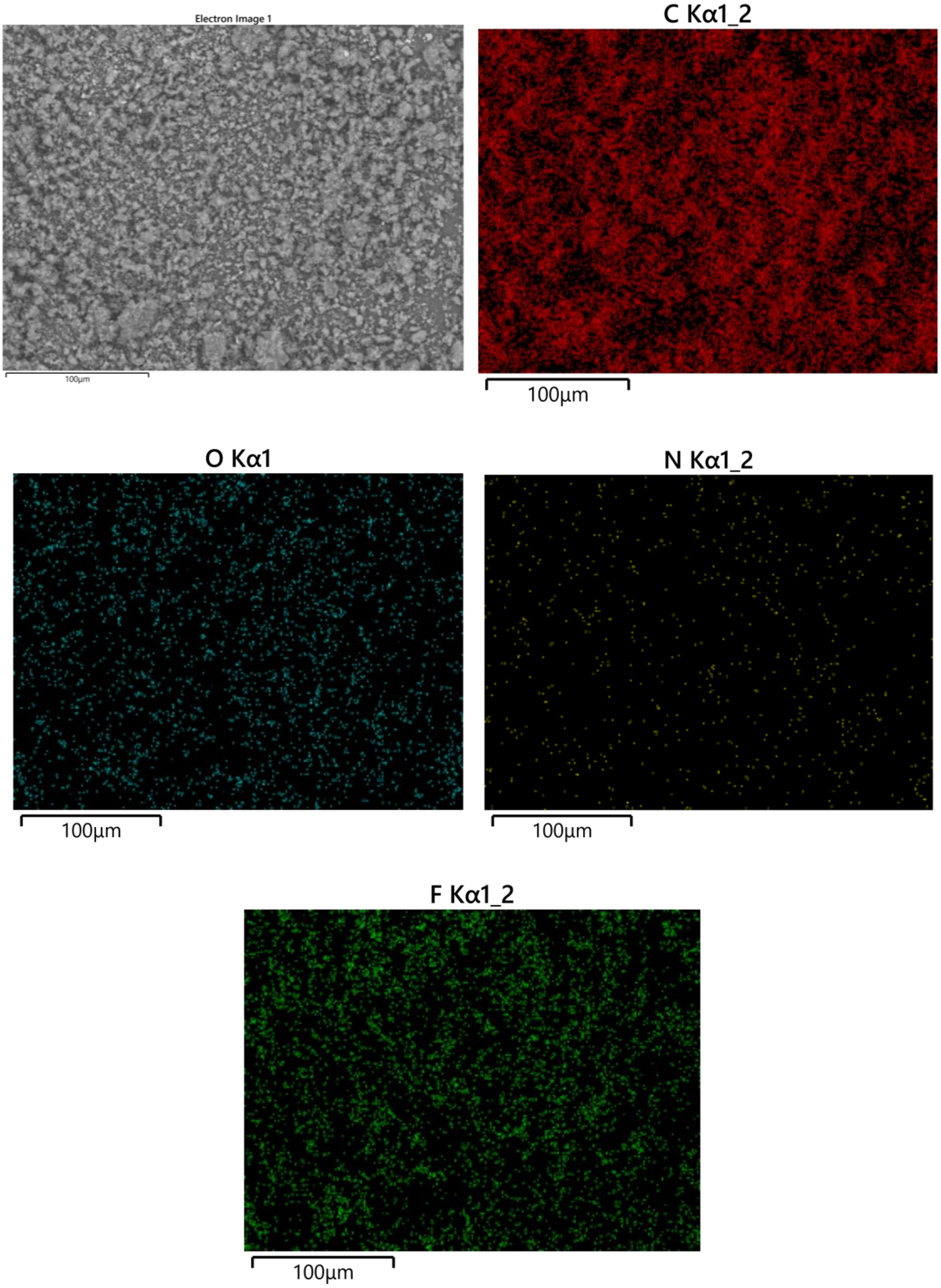

Figure S36. EDX mapping of the elements C (*red*), O (*blue*), N (*yellow*) and F (*green*) for a sample of **PCP-PI5**.

7 References

1. Myers, A. L.; Prausnitz, J. M., Thermodynamics of mixed-gas adsorption. *AIChE Journal* **1965,** *11* (1), 121-127.

2. Lee, S.; Lee, J. H.; Kim, J., User-friendly graphical user interface software for ideal adsorbed solution theory calculations. *Korean Journal of Chemical Engineering* **2018,** *35* (1), 214-221.

3. Zheng, Z.; Zhang, M.; Wen, Y.; Wang, Z.; Yan, J., Synthesis and gas separation performance of intrinsically microporous polyimides derived from 9, 9-bis (3, 4-dicarboxyphenyl) fluorene dianhydride. *European Polymer Journal* **2025**, 114301.
